# Early prediction of antigenic transitions for influenza A/H3N2

**DOI:** 10.1371/journal.pcbi.1007683

**Published:** 2020-02-18

**Authors:** Lauren A. Castro, Trevor Bedford, Lauren Ancel Meyers

**Affiliations:** 1 Department of Integrative Biology, The University of Texas at Austin, Austin, Texas, United States of America; 2 Analytics, Intelligence, and Technology Division, Los Alamos National Laboratory, Los Alamos, New Mexico, United States of America; 3 Vaccine and Infectious Disease Division, Fred Hutchinson Cancer Research Center, Seattle, Washington, United States of America; 4 Santa Fe Institute, Santa Fe, New Mexico, United States of America; University of Zurich, SWITZERLAND

## Abstract

Influenza A/H3N2 is a rapidly evolving virus which experiences major antigenic transitions every two to eight years. Anticipating the timing and outcome of transitions is critical to developing effective seasonal influenza vaccines. Using a published phylodynamic model of influenza transmission, we identified indicators of future evolutionary success for an emerging antigenic cluster and quantified fundamental trade-offs in our ability to make such predictions. The eventual fate of a new cluster depends on its initial epidemiological growth rate––which is a function of mutational load and population susceptibility to the cluster––along with the variance in growth rate across co-circulating viruses. Logistic regression can predict whether a cluster at 5% relative frequency will eventually succeed with ~80% sensitivity, providing up to eight months advance warning. As a cluster expands, the predictions improve while the lead-time for vaccine development and other interventions decreases. However, attempts to make comparable predictions from 12 years of *empirical* influenza surveillance data, which are far sparser and more coarse-grained, achieve only 56% sensitivity. By expanding influenza surveillance to obtain more granular estimates of the frequencies of and population-wide susceptibility to emerging viruses, we can better anticipate major antigenic transitions. This provides added incentives for accelerating the vaccine production cycle to reduce the lead time required for strain selection.

## Author summary

The efficacy of annual seasonal influenza vaccines depends on selecting the strain that best matches circulating viruses. This selection takes place 9–12 months prior to the influenza season. To advise this decision, we used an influenza A/H3N2 phylodynamic simulation to explore how reliably and how far in advance can we identify strains that will dominate future influenza seasons? What data should we collect to accelerate and improve the accuracy of such forecasts? And importantly, what is the gap between the theoretical limit of prediction and prediction based on current influenza surveillance? Our results suggest that even with detailed virological information, the tight race between the antigenic turnover dynamics and the vaccine development timeline limits early detection of emerging viruses. Predictions based on current influenza surveillance do not achieve the theoretical limit and thus our results provide impetus for denser sampling and the development of rapid methods for estimating viral fitness.

## Introduction

Seasonal influenza A/H3N2 causes significant annual morbidity and mortality worldwide, as well as severe economic losses [1]. In the United States, the 2017–2018 season was unusually long and severe, lasting over 16 weeks and causing over 900,000 hospitalizations and 80,000 fatalities, including 183 pediatric deaths [2–4]. The global health community continually tracks H3N2 and annually updates the H3N2 component of the seasonal influenza vaccine. However, annual influenza epidemics continue to impart a significant public health burden. The rapid antigenic evolution of the influenza virus via mutations in hemagglutinin (HA) glycoproteins and neuraminidase (NA) enzymes [5,6], and logistical requirement of selecting vaccine strains almost a year prior to the flu season pose a significant challenge. Vaccines target the antigen-binding regions of dominant influenza subtypes. While a particular subtype may circulate for a few years, strong positive selection for new antigenic variants will eventually produce antigenic drift [7–9], rendering a vaccine less effective if new mutations in the antigen-binding regions are not included in vaccine chosen strains [10,11]. The typical reign of a dominant subtype ranges from two to eight years [12,13]. A meta-analysis of test-negative design studies found that the H3N2 component of the seasonal flu vaccine had an estimated average efficacy of 33% (CI = 26%-39%) from 2004–2015 [14].

The World Health Organization’s Global Influenza Surveillance and Response System (GISRS) coordinates influenza surveillance efforts to survey and characterize the diversity of influenza viruses circulating in humans. Viral samples are rapidly analyzed via sequencing of HA and NA genes, serologic assays, and other laboratory tests to identify newly emerging antigenic clusters. Within the past decade, the number of complete HA gene sequences in the GISAID EpiFlu [15,16] database has increased tenfold, from fewer than 1,000 in 2010 to over 10,000 in 2017 [17]. Molecular data at high spatiotemporal resolution could potentially revolutionize influenza prediction. However, the research and public health communities have just begun to determine effective strategies for extracting and integrating useful information into the vaccine selection process.

Phylodynamic models describe the interaction between the epidemiological and evolutionary processes of a pathogen [18]. The availability of molecular data coupled with the recent development of detailed, data-driven phylodynamic models has galvanized the new field of viral predictive modeling [19–22]. These models aim to predict the future prevalence of specific viral subtypes based on past and present molecular data. For example, one approach generates one-year ahead forecasts of clade frequency using a fitness model parameterized by the number of antigenic and genetic mutations that dictate the virus’ antigenicity and stability respectively [23]. Another method maps antigenic distance from hemagglutination inhibition (HI) assay data onto an HA genealogy to determine whether the changes in antigenicity among high-growth clades necessitate a vaccine composition update [24]. A third model predicts which clade will be the progenitor lineage of the subsequent influenza season by estimating fitness using a growth rate measure derived from topological features of the HA genealogy [25]. All three approaches have been tested on historical predictions. Łuksza’s & Lässig’s model [23] predicted positive growth for 93% of clades that increased in frequency over one year. Steinbruk *et al*. [24] predicted the predominant HA allele over nine influenza seasons with an accuracy of 78%. Both Łuksza’s & Lässig’s [23] and Neher *et al*. [25] model predictions of progenitor strains to the next season’s performed similarly. Since 2015, both these models have been used to provide recommendations on vaccine composition for the upcoming influenza seasons [26–28].

Taken together, this body of work points to the promise of predictive evolutionary models. Phylodynamic simulation models provide a complementary window into the molecular evolution of emerging viruses. By observing influenza evolution *in silico*, we can take a rigorous experimental approach to test hypotheses about early indicators of cluster [29,30] success and design surveillance strategies to inform vaccine strain selection. Here, we simulate decades of H3N2 evolution and transmission using a published phylodynamics model [31,32] and analyze the simulated data to identify early predictors of a cluster’s evolutionary fate. Viral growth rates––both for an emerging cluster and its competitors––are the most robust predictors of future ascents. When a new antigenic cluster first appears at low frequency (e.g., 1% of sampled viruses), our statistical logistic regression models can predict whether it will eventually rise to dominance (e.g., maintain a relative frequency greater than 20% of sampled viruses for at least 45 days) with reasonable confidence and advanced warning. We also attempt to adapt these statistical models into actionable guidelines for global influenza surveillance by developing proxy indicators that can be readily estimated from available data. Using both simulated data and 6,271 influenza sequences collected between 2006 and 2018, we quantify the limits in the accuracy, precision and timeliness of predictions, and construct models to predict future frequencies of emerging clusters.

## Results

Our simulations roughly reproduced the global epidemiological and evolutionary dynamics of H3N2 influenza over a 25-year period. Without seasonal forcing, prevalence rose and fell, peaking every 3.2 years on average (s.d. = 1.6). These dynamics reflected the turnover and competition of antigenic clusters. The median of the most recent common ancestor (TMRCA) in our simulations was 5.9 years (IQR 4.62–7.9), which is higher than empirical estimates of 3.89 years [13]. The median life span of established clusters was 1128 days (s.d. = 480), corresponding to roughly 3.5 years. However, the annual incidence of influenza in our model (4.0%, 95% CI 0.37–9.7%) was lower than empirical annual incidence estimates of 9–15% [13]. Given the model only simulated the transmission of H3N2 and not all circulating influenza types, our annual incidence was comparable to empirical estimates [33].

We assumed that clusters become detectable once they cross a relative frequency threshold of 1% and were fully established if they maintained a relative frequency above 20% for at least 45 weeks. In our simulations, 2% of the approximately 200 novel antigenic clusters per year overcame early stochastic loss to reach detectable levels. As the relative frequency of a newly emerging cluster increased, the probability that the cluster will ultimately establish also increased. There was an inverse relationship between the number of clusters that reached a threshold and the probability of future success. For example, far fewer clusters reached a relative frequency of 10% than 1%. If a cluster succeeded in reaching relative frequency thresholds of 1%, 6%, and 10%, its probability of establishing increased from 13% to 50% to 67% (S3 Fig).

**Fig 1:**
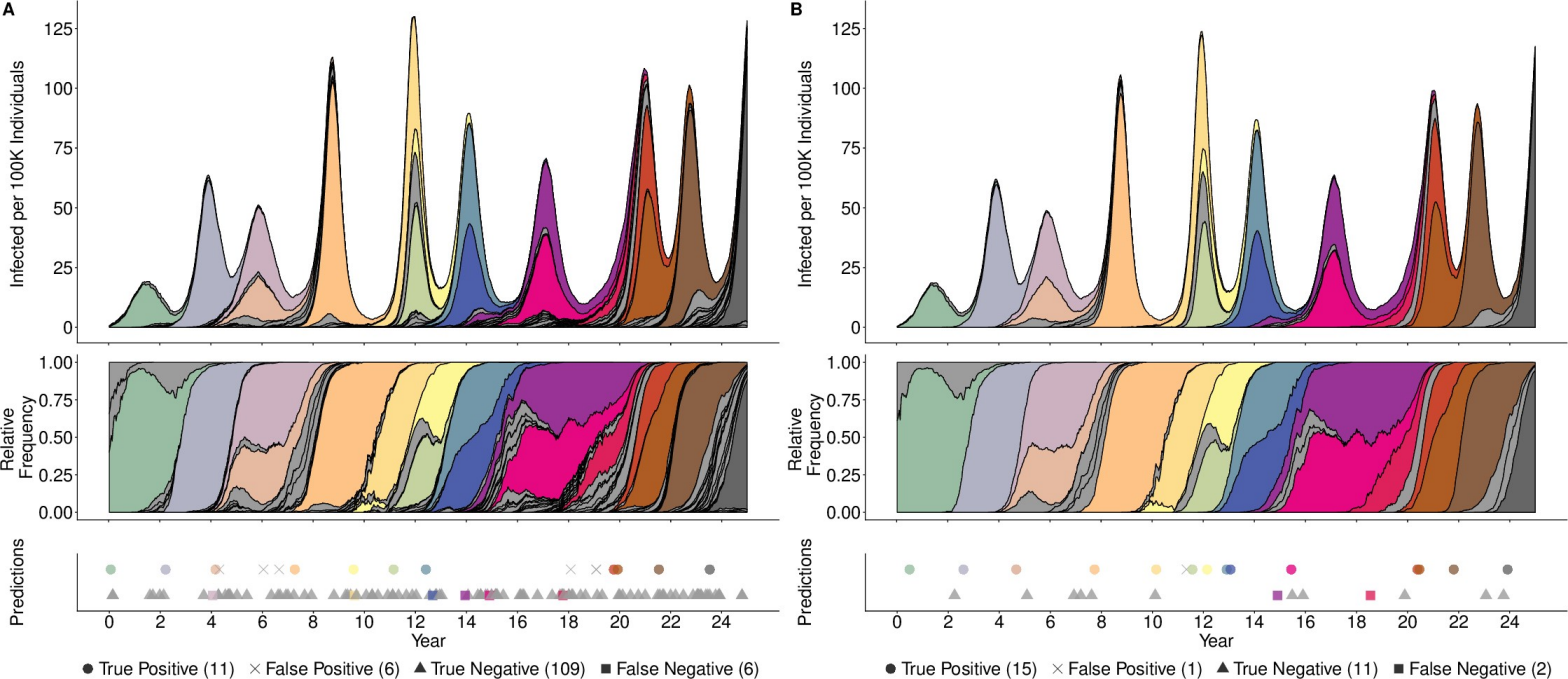
**Out-of-sample predictions of antigenic cluster evolutionary success at relative frequency thresholds of 1% (A) and 10% (B).** Grey shading indicates clusters that surpass the surveillance threshold, but do not establish. Other colors correspond to distinct antigenic clusters that eventually establish. The top time series graphs depict the absolute prevalence of antigenic clusters; the middle graphs give their relative frequencies. The bottom panels indicate the timing and accuracy of out-of-sample predictions based on the optimized model for each surveillance threshold. The top row of symbols indicate clusters predicted to succeed, with true positives indicated by circles and false positives indicated by crosses; the bottom row indicates clusters predicted to circulate only transiently, true negatives indicated by triangles and false negatives indicated by squares. The number of predictions in each category is provided in the legend.

Our logistic regression model classified clusters as either *positives* that are likely to establish or *negatives* that are expected to circulate only transiently. As we increased the surveillance threshold, the fraction of successful clusters that were misclassified as negatives decreased. In a representative out-of-sample 25-year simulation, 17 of 132 detectable clusters eventually rose to dominance (Fig 1). Of these, 65% and 88% were correctly predicted when they reached the 1% and 10% surveillance threshold, respectively. The number of true negative events decreased considerably, from 109 at the 1% surveillance threshold to only 11 at the 10% surveillance threshold, while the other types of events held relatively constant.

Across all surveillance thresholds, the first four predictors chosen through forward model selection were predictors that captured levels of antigenic and genetic novelty in the focal cluster and background viral population, namely the relative growth rate of the focal cluster (*R*_c_/〈*R*〉), the background variance (var(*R*)) and mean (〈*R*〉) of viral growth rates, and the relative deleterious mutational load of the focal cluster (*k*_*c*_/〈*k*〉). Population-level epidemiological quantities were only selected for models at low surveillance thresholds (2–4%); in these models, overall prevalence had a slightly negative correlation with future viral success (Table 1). The median number of predictors chosen was 6.5, with a range of 5 to 7. The best fit models are described in S2 Table.

**Table 1:** Predictors selected by five-fold cross validation and forward selection. The top four variables were selected in the identical order (as listed) across all surveillance threshold models. The fifth predictor, relative variance in transmissibility, was included in all models, but not always as the fifth chosen. In the formulas, c refers to cluster-level quantities. The rightmost column gives the full range of fitted coefficients (log-odds) across all models based on the five-fold cross validation for each surveillance thresholds’ final model. *var(*σ*_*c*_) was calculated across all hosts; var(*S*_*eff*_) was calculated across only infected hosts. *I* = number of infected hosts, *N* = total number of hosts, *σ*_*c*_ = effective susceptibility to infection by cluster c, *β*^*k*^ = the transmission rate of the virus carrying *k* deleterious mutations. Formulas to calculate each quantity are in S1 Table.

	Predictor	Symbol	Models Included (Surveillance Threshold %)	Coefficient Estimate
All Models	1.Relative growth rate	*R*_*c*_/〈*R*〉	1–10	[2.3, 2.64]
	2.Variance in population R	var(*R*)	1–10	[-0.72, -0.49]
	3.Population R	〈*R*〉	1–10	[0.32, 0.42]
	4.Relative mutational load	*k*_*c*_/〈*k*〉	1–10	[-0.34, -0.21]
Some Models	Relative variance in transmissibility	var(*β*_*c*_)/var(*β*)	1–10	[0.17, 0.34]
	Variance in susceptibility to cluster c	var(*σ*_*c*_)	1–6	[0.16, 0.20]
	Frequency of current dominant cluster	*I*_*c*_/*I*	3,5,8,9	[0.14, 0.21]
	Proportion of individuals infected	*I*/*N*	2	-0.17
	Total number of individuals infected	I	3,4	[-0.17, -0.16]
	The most recent common ancestor	tMRCA	10	-0.16
	Relative variance in susceptibility*	var(*σ*_*c*_)/var(*S*_eff_)	1	[0.12, 0.16]

**Fig 2:**
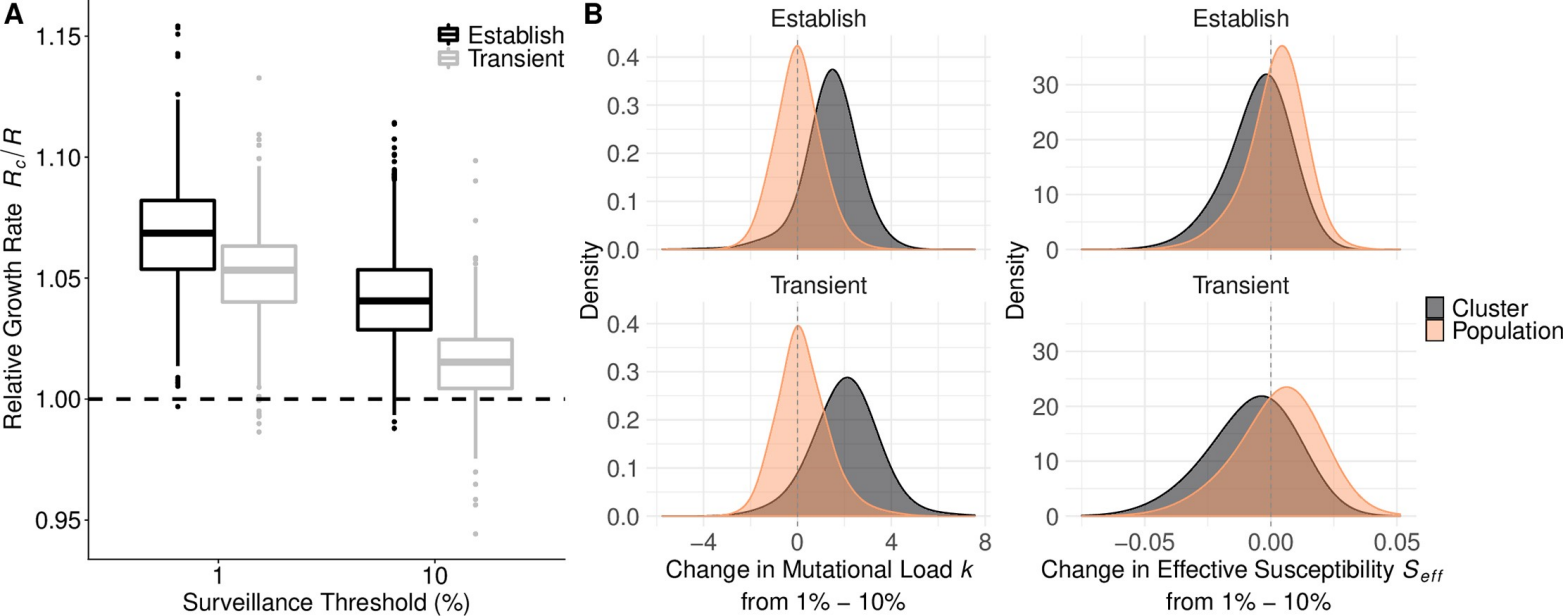
Relative growth rates predicted future success. (A) Clusters that eventually establish had significantly higher *R*_c_/〈*R*〉 than those that fail to establish. As clusters increased in relative frequency from 1% to 10%, their *R*_c_/〈*R*〉 generally declined but the distinction between future successes and future failures became more pronounced. (B) *R* is a composite value based on the mutational load and *S*_eff_. We compared the mutational load (left) and *S*_eff_ (right) of a cluster when it crossed the 1% and 10% thresholds by subtracting the former from the latter (orange distributions); we simultaneously calculated the difference in average mutational load and *S*_eff_ across the entire viral population (grey distributions). The top and bottom rows show the distributions of change for clusters that establish and transiently circulate, respectively. The decrease in a cluster’s fitness advantage was driven by both increasing mutational load and a decreasing *S*_eff_. The background mutational load did not change noticeably, while the background *S*_eff_ increased slightly.

We examined the dynamics of the top two predictors. As newly emerging clusters rose in relative frequency from 1% to 10%, their relative growth rate declined towards one. That is, they approached the population average fitness (Fig 2). The relative growth rate was significantly higher for clusters that will eventually establish than those that will burn out, with the separation between the two groups increasing as the clusters ascend in frequency (Fig 2A). This predictor is a composite quantity, estimated based on both mutational load and effective susceptibility. We compared these two quantities at two time points, when the clusters reached 1% and 10% frequencies. Mutational load increased and effective susceptibility decreased in ascending clusters, with more extreme changes occurring in clusters that ultimately failed to establish. We also measured the changes in these two quantities for the entire population, and found that the background mutational load remained relatively constant and background effective susceptibility increased slightly. The background effective susceptibility peaked when a new cluster began to constitute a major proportion of the circulating types––at this point the immunity from previous infections was not strongly protective against the newly dominant cluster. The decline in cluster fitness likely stems from the accumulation of deleterious mutations and exhaustion of the susceptible population (Fig 2B). While this occurred within both established and transient clusters, the mutational loads in established and transients increased by averages of 1.4 and 2.04 mutations, respectively (Wilcox, p < 2.2e-16).

**Fig 3:**
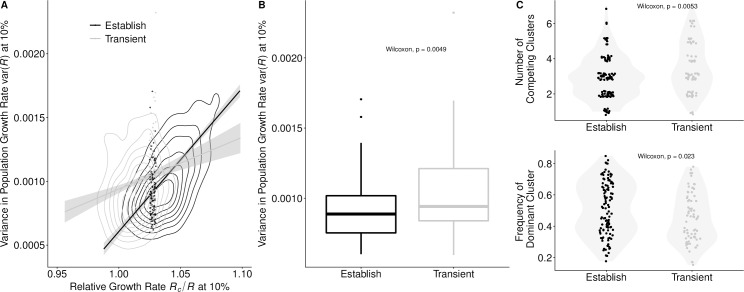
Viral competition predicted future success for clusters with borderline growth rates. (A) Clusters with only a slight *R*_c_/〈*R*〉 advantage were more likely to establish if the background var(*R*) was low. Clusters with higher *R*_c_/〈*R*〉 were successful regardless of var(*R*). Contour lines indicate the density of values of *R*_c_/〈*R*〉 and var(*R*). The lines represent the correlation between the variables for successful and transient clusters. (Success-black: r = 0.63, p < 2.2e-16; Transient-grey: r = 0.18, p <1.2e-06). The dots represent clusters with *R*_c_/〈*R*〉’s between 1.025–1.030, a range within the individual distributions of *R*_c_/〈*R*〉 for success and transient clusters that do not statistically differ (Wilcox, p = 0.4551). (B) For clusters falling within this ambiguous range of *R*_c_/〈*R*〉, var(*R*) was significantly higher in transient clusters than in established clusters, and (C) in comparison to transient clusters, successful clusters tended to face fewer co-circulating clusters (Wilcox, p = 0.0053), with the current dominant cluster at higher frequency (Wilcox, p = 0.023). Points represent the number of circulating clusters and the frequency of the dominant cluster; shading represents the kernel density estimation of the distribution of points. Across all graphs, values were calculated when the focal clusters reach a 10% surveillance threshold.

The background variance in viral growth rates, var(*R*), was the second most informative predictor. The lower the variance, the more likely a cluster was to establish. However, it was a weaker predictor than *R*_c_/〈*R*〉’s; the estimated logit coefficient of the *R*_c_/〈*R*〉 was approximately four times that of var(*R*) (Table 1). The var(*R*) tended to increase as a cluster expanded from 1% to 10% relative frequency (Wilcox, p < 2.2e-16). This may stem from diverging fitnesses of the newly expanding cluster and the receding dominant cluster, which had likely accumulated a considerable deleterious load and burned through much of its susceptible host population. A higher var(*R*) decreased the probability of a cluster being successful, particularly when a cluster had only a modest growth rate. Clusters with high *R*_c_/〈*R*〉’s were successful even when emerging in highly variant environments (Fig 3A). High variance may reflect high levels of inter-viral competition. If we considered both transient and established clusters with similar *R*_c_/〈*R*〉 (ranging from 1.025 to 1.03), successful clusters encountered significantly fewer co-circulating clusters, and the frequency of the resident dominant cluster was significantly higher (Fig 3C). This may reflect suppression of competition by the dominant cluster, creating a vacuum for a moderately fit cluster to fill.

**Fig 4:**
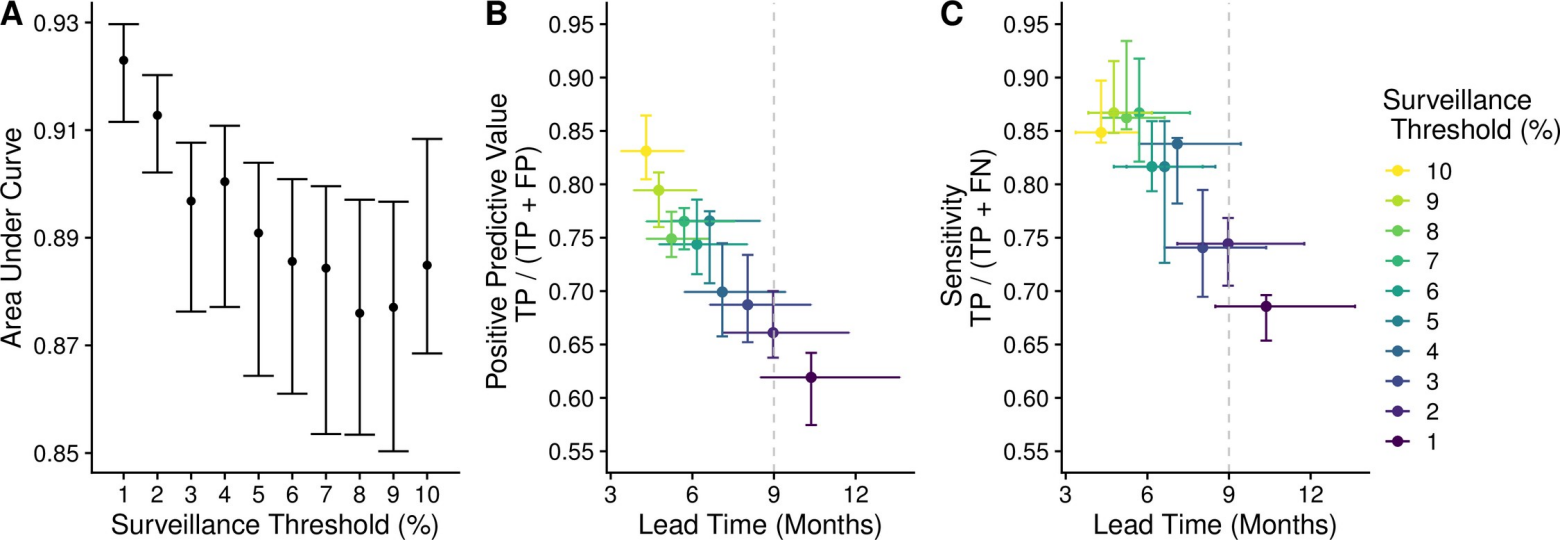
Model performance across surveillance thresholds. (A) Area under the receiver operator curve (AUC) suggests that models can predict successful from unsuccessful clusters by the time they reached 1% of circulating viruses, with discriminatory power declining slightly as clusters rose in frequency. Bars represent the max, median, and minimum AUC values across 5-fold cross validation. (B-C) There was a trade-off between lead time and model performance. The horizontal bars represent the IQR of time between the moment the expanding antigenic cluster reaches the surveillance threshold and when it reaches the success criteria. Vertical bars represent the range and median positive predictive value (B) and sensitivity (C), across five-fold evaluation. Colors correspond to the best fit model for each surveillance threshold. Dashed gray lines indicate lead times of nine months, which represents the current time between the Northern Hemisphere vaccine composition meeting in February and the following start of the influenza season in October.

When forecasting influenza dynamics, there may be tradeoffs between prediction certainty, the extent advanced warning, and the surveillance effort required to detect and characterize emerging viruses. Across our ten models, there was a marked trade-off between lead-time and reliability, with low surveillance thresholds providing earlier but less accurate indication of future threats (Fig 4). Across simulations, the median time difference between a cluster reaching the 1% and 10% surveillance thresholds was approximately 7 months (IQR: 154–294 days).

Classifier models had substantial discriminatory and predictive power even when an antigenic cluster was present at low frequencies (Fig 4A). Model AUC’s tended to decrease as the frequency of the candidate clusters increased. Conversely, the positive predictive value (PPV) and sensitivity increased at higher surveillance thresholds. The gains in sensitivity and PPV per month decreased at higher surveillance thresholds. Between the 1% and 5% surveillance thresholds, there was on average a 4% increase in sensitivity and a 4.5% increase in positive predictive value per month lost in lead-time. However, between the 6% and 10% surveillance thresholds, sensitivity gains dropped to 1.2% and PPV to 3.6% per month lost in lead-time. This decreasing tradeoff between gain in certainty and loss of lead-time reflected shorter intervals between surveillance thresholds as the cluster began to rapidly expand and the model’s prediction capabilities reached upper capacity.

### Restricting surveillance knowledge

We next considered an alternative surveillance paradigm. Rather than waiting for specified surveillance thresholds, we fit models to predict the presence and frequency of clusters based on opportunistic sampling of clusters, Cluster frequencies tended to skew towards low frequencies (S6 Fig). Our best fit model for predicting the future success of all clusters present at a random time point performed comparably to our best models for low surveillance thresholds (S7 Fig). We next fit a second two-part model that sequentially predicted the presence-absence and the frequency of a cluster in three-month intervals out to one year ahead (S8 Fig). The model predicted up to twelve-month ahead presence-absence with 92% discriminatory power (AUC). However, the accuracy of the frequency predictions declined after six months, with a tendency to underestimate the frequencies of future dominant clusters (S4 and S5 Tables). The top predictors included the frequency of the cluster at the time of sampling and most of the top predictors selected for the surveillance threshold models.

The primary predictor across all models––the relative growth rate of a cluster––cannot easily be estimated from available surveillance data. Thus, we built and evaluated bivariate logistic regression models on simulated data that predict future success using more easily attained proxies (Table 2). One model considered the time taken for the cluster to rise from 6% to 10% relative frequency and the total number of clusters that grew during this period; the other considered the fold-change in the relative frequency of the cluster between these time points and the background variance in fold-change. Of the four proxies, all but the relative fold-change of the cluster were statistically significant predictors, with negative effects on the probability of cluster success (S4 Fig). These resulting models had higher sensitivity than positive predictive values, but both sensitivity and positive predictive value were lower in these models than the model using the complete simulated data at the 10% surveillance threshold. We also tested analogous models using statistics calculated at alternative surveillance checkpoints (1% to 5%, 3% to 5%, and 8% to 10%), and found that the 6%-10% comparison performed best (S3 Table).

**Table 2:** Performance of proxy predictors at the 10% surveillance threshold. Model 1 predicted the fate of a cluster using the top two predictors in our best fit model. The two proxy models used data from two time points, when the cluster reached relative frequencies of 6% (*t*_1_) and 10% (*t*_2_). Model 2 considered the time elapsed between and the number of competing expanding clusters. Model 3 considered the relative fold change in the focal cluster between the two time points and the population-wide variance in fold change. Performance values are the median of five-fold cross-validation.

Model	Type	AUC	PPV	Sensitivity
1. *R*_c_/〈*R*〉+var(*R*)	Actual	0.88	0.81	0.89
2. $\delta_{c}(t_{1},t_{2})+N_{\Delta_{j}(t_{1}t_{2})>1}$	Proxy	0.78	0.74	0.87
3. *χ*_c_(*t*_1_,*t*_2_)+var(Δ_*j*_(*t*_1_*t*_2_))	Proxy	0.67	0.66	0.95

### Application to real-world scenarios

**Fig 5:**
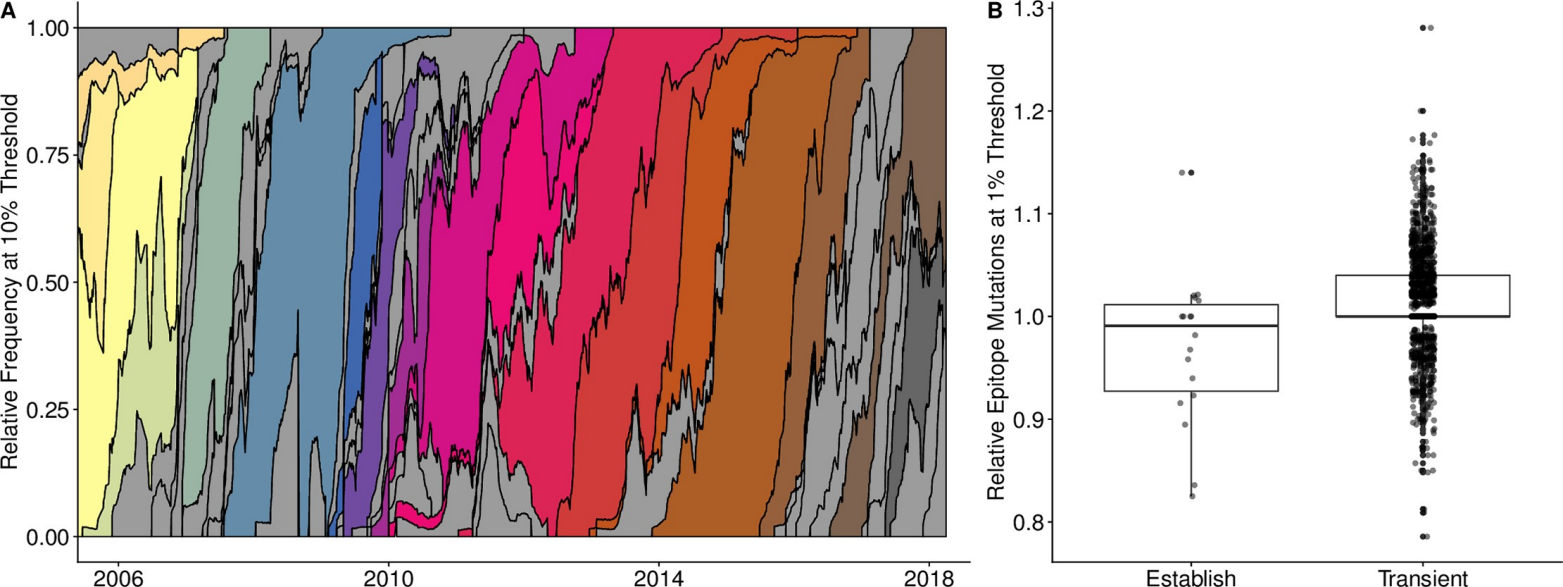
Empirical antigenic dynamics of influenza A/H3N2, 2006–2018. (A) Relative frequencies of all antigenic clusters that reached the threshold of at least 10% of sampled viruses. Frequencies were calculated using a 60-day sliding window. Grey shading indicates clusters that surpassed the 10% threshold, but did not eventually establish (i.e., reached relative frequency of at least 20% for at least 45 days). Other colors indicate distinct antigenic clusters that eventually established. (B) A low relative number of epitope mutations when a cluster reached the 1% relative frequency threshold was an early indicator of future success (Holm’s P. adjusted, p < 0.003). We divided the number of epitope mutations of a focal cluster by the average number of mutations of simultaneously circulating clusters.

Finally, using 6271 real influenza A/H3N2 sequences sampled from around the globe between 2006 and 2018, we assessed whether this methodology can use available influenza surveillance data to predict emerging clusters. Using the opportunistic sampling nature of the data, our models classified clusters that had reached at least 1% relative frequency, but not yet 20% relative frequency as either likely to establish or as expected to circulate only transiently. Clusters were distinguished by single mutations to epitope sites on the HA1 sequence and successful clusters were those that reached a relative frequency of at least 20% for at least 45 days. Despite sparse sampling, the dynamics of antigenic transitions resembled those produced by our simulations (Fig 5A). Over the 12-year period, dominant clusters circulated for an average of 2.25 years (s.d. 1.17); 44 clusters reached a relative frequency of 10%; 18 of the 44 were eventually successful.

We could not directly estimate the growth rate of each emerging cluster from available data (as in Table 1). Thus, the epitope mutations in the empirical data were a proxy for the antigenic mutations that were tracked in the simulated data. However, while in the simulated data we could capture the immune escape effect associated with each antigenic mutation, in the empirical data, we could only note presence-absence of epitope mutations. Since growth rate advantages may stem from mutations in the epitope region of the virus and such mutations can be readily identified and counted using available sequence data, we evaluated the presence of epitope mutations as a possible proxy for growth rate. Specifically, we divided the average number of epitope mutations in viruses within a given cluster by the average number found in other co-circulating viruses. For clusters that reached at least 1% relative frequency, this quantity was less than one for clusters that eventually established (N = 18) and greater than one for transient clusters that did not establish (N = 1516); this difference was statistically significant (Holm’s P. adjusted, p = 0.03) (Fig 5B). As noted, the absolute number of epitope mutations does not capture the effect on immune escape. A possible explanation for why emerging clusters had fewer epitope mutations is that individual mutations of large fitness effects provided a competitive advantage over multiple mutations of smaller effect. However, this difference was not significant when measured for clusters reaching at least the 2% relative frequency surveillance threshold (Holm’s P. adjusted, p = 0.18). Therefore, single mutations of large effect are most influential in overcoming the initial stochastic hurdle to emergence.

We fit classifier models to the empirical data using relative epitope number and other proxies for fitness (e.g. fold change and growth rate between sequential sampling of a cluster) and competition, (e.g., the number of co-circulating clusters) (S6 Table) for all clusters that reached at least 1% relative frequency. Given the limited data, we estimated fitness and competition proxies using the first two sample points for each cluster that had not yet reached 20% relative frequency. Several of these proxies were able to predict future evolutionary success. However, our best two-predictor model, which considered a cluster’s frequency at its first sample point and the number of competing clusters lost by the second sample point, achieved only a sensitivity of 56% and PPV of 50% at predicting the evolutionary fate of a cluster (S7 Table). Both sensitivity and positive predictive values were below those of the simulated-based models built on the 1% surveillance threshold (69% and 62% respectively) and the simulated-based models built on an opportunistic sampling scheme that more closely reflected the structure of the empirical data (75% and 70% respectively, S7 Fig).

## Discussion

Until we develop an effective universal flu vaccine, seasonal vaccines will remain the frontline of influenza prevention. The severe 2017–2018 influenza season was a stark reminder that anticipating dominant strains with sufficient lead time for incorporation into vaccines is paramount to public health. Here, we analyzed over 1500 years of simulated influenza phylodynamics to explore the predictability of antigenic emergence and identify early predictors of future evolutionary success that can be plausibly monitored via ongoing surveillance efforts. We compared our results derived from a detailed omnipotent simulation to a simulated scenario that places restrictions on the data available for virus characterization and to an empirical dataset of influenza sequences from 2006–2018.

Phylodynamic models provide insight into both the interplay of evolutionary and epidemiological processes and how these dynamics are manifested in observable data. In simulated data, the strongest predictor of future dominance across all of our models is the relative effective reproductive number of a cluster, that is, the growth rate of the cluster compared to the average growth rate across the viral population. This measure of viral fitness incorporates both the real-time competitive advantage (vis-a-vis the immunological landscape) and deleterious mutational load. Intuitively, faster growing clusters are more likely to persist and expand. Our ability to predict the fate of an emergent virus improves as the cluster increases in relative frequency. Both sensitivity––the proportion of successful clusters detected by the model––and positive predictive value––the proportion of predicted successes that actually establish––surpass 80% by the time a cluster has reached 10% relative frequency.

The second most informative predictor selected across all models––the population-wide variance in the effective growth rate, var(*R*)––requires a more nuanced interpretation. The greater the background variance at the time a cluster is emerging, the less likely the cluster is to succeed. To unpack this result, we analyzed the competitive environment of emerging clusters with only modest growth rates; rapidly growing clusters are likely to succeed regardless of their competition. Within this class of slowly emerging viruses, those that initially face a single high frequency dominant cluster and fewer co-emerging competitors are more likely to succeed [34,35]. A recent sweep by a dominant cluster leaves a wake of immunity that can be exploited by antigenically-novel clusters that stochastically battle for future dominance. We hypothesize that these two conditions––a reigning dominant cluster and reduced competition with emerging novelty––reduce the overall variance in viral growth rate and explain the negative correlation between this quantity and the future ascent of an emerging cluster.

Our top predictors of viral emergence require a comprehensive sampling of the viral and host population. Although exact measurements of these quantities are practically infeasible, our results suggest that targeting molecular surveillance towards precise and accurate estimation of viral growth rates, both for newly emerging clusters and the resident circulating viruses, may enhance influenza prediction. One approach is to target the two key components of growth rate separately––mutational load and effective susceptibility. Changes in the mutational load can be estimated from sequence data, comparing the number of differences that occur in non-epitope portions of the genome over time [23,27,36]. Our parameterization of *R*_c_ follows the empirical method of [23], with fitness costs based on nonsynonymous amino acid differences between a given strain and its most recent common ancestor. Estimating the effective susceptibility is more challenging, as it depends on the interaction between an individual’s exposure history [37–39] and new amino acid substitutions in epitope coding regions [6,40]. Nonetheless, several studies introduce innovative methods for estimating susceptibility from the historic distribution of influenza subtypes, seasonal influenza prevalence, and HI-titers. For example, Neher *et al*. [36] predict antigenic properties of novel clades by mapping both serological and sequence data to a phylogenetic tree structure of HA sequences. Łuksza & Lässig estimate effective susceptibility by first estimating the historic frequency of clades in six-month intervals and then estimating cross-immunity between those clades and the focal cluster based on amino acid differences in epitope regions [23]. However, both methods only consider clusters that have already surpassed 10% relative frequency, at which point strains are thought to be geographically well-mixed and less prone to geographic sampling bias.

Another approach to estimating the growth rate of an emerging cluster is to treat it as a composite quantity. We evaluated several proxy measures of cluster growth rate, including the relative fold-change in frequency between two time points. Models based on fold change rather than the true growth rate actually have greater sensitivity, that is, they are more likely to detect clusters destined for dominance when they first emerge. However, the positive predictive values of our best models drop from 0.81 to 0.67, meaning that replacing the true growth rates with an approximation increases the rate of false alarms. Importantly, the proxy model improves with the addition of a second predictor, the variance in fold-change across the viral population, which can also be readily estimated from surveillance data. Thus, variance in fitness appears to be a robust secondary predictor of future sweeps, regardless of how fitness is quantified. Surprisingly, a model based on seemingly naive approximations of growth rate––the time elapsed between two frequency thresholds and the number of other co-circulating clusters rising in frequency––was even more accurate, though still inferior to the true growth rate models. We did not evaluate a promising alternative strategy for approximating fitness, based on the phylogenetic reconstruction of currently circulating sequences [25,41]. Unlike the proxies we considered, this does not require historical data but does rely on pathogen sequencing. Finally, although not as informative as predictors that quantify the evolutionary and immunological state of the population, easily quantifiable predictors such as the total number of infected individuals or the frequency of the circulating dominant cluster, can be incorporated into future predictive models.

Our attempts to apply the optimized models to empirical data were of limited success. While the global evolutionary dynamics of influenza A/H3N2 clusters visually resemble those observed in our simulations, the sparse genotypic data available do not permit estimation of the phenotypic predictors identified in our study. In the absence of these phenotypic predictors, we identified proxies of fitness and competition in the number of epitope mutations and frequency. The number of epitope mutations in a newly emerging cluster relative to co-circulating viruses provides early indication of future success. This provides proof of concept that the evolutionary viability of influenza viruses is predictable but will require better models for estimating viral fitness from sequence data and the expansion of surveillance efforts [42] to collect phenotypic data reflecting the mutational loads of viruses and dynamic trends in population susceptibility early in their circulation. The poor model performance likely stems from both data quality and data quantity. At the 10% surveillance threshold, we trained and tested each *simulation* model on an average of 1488.8 antigenic clusters (s.d. 23.79) and 372.2 clusters (s.d. 23.79), respectively. In contrast, the *empirical* models were trained and tested on 303 and 75 clusters (only three of which eventually established), respectively. The empirical models may also be limited by real-world complexity that is not captured in the simulation model. Whereas the model assumes a well-mixed population of 40 million people, actual influenza circulation occurs in a larger and more heterogeneous population in which the fate of a newly emerging virus may be far more context dependent and thus less predictable.

Nonetheless, we believe that our simulation results provide robust insight into the limits of influenza surveillance and molecular forecasting. While the certainty of our predictions improves as clusters increase in relative frequency, there is a trade-off with lead time. The longer we wait to assess a rising cluster, the less time there will be to update vaccines and implement other intervention measures. For a successful cluster detected at a relative frequency of 1%, there will be, on average, 10 months before the cluster becomes established (maintains a relative frequency over 20% for 45 days). If detected only after reaching a relative frequency of 10%, the expected lead time shrinks to four months. Although real-world surveillance is noisy and dependent on sufficient sampling depth and geographic coverage, our results suggest that, with a perfect knowledge of the host and viral populations, predictions can be made with at least 85% sensitivity and confidence before a cluster rises to 10% of all circulating strains.

As policy-makers consider new strategies for antigenic surveillance and forecasting, the trade-off between prediction accuracy and lead time has practical implications. For example, a detection system targeting new viruses as soon as they reach 1% relative frequency has the benefit of early warning and drawback of low accuracy, which translate into economic and humanitarian costs and benefits. On the positive side, early warning increases the probability that seasonal vaccines will provide a good match with circulating strains, and thus lowers the expected future morbidity and mortality attributable to seasonal influenza. Based on the vaccine production and delivery schedule, the surveillance window for emerging clades is from October to February for the Northern Hemisphere and vaccine composition is determined at an international meeting in February [17]. Our analysis suggests that, at nine months before an emerging cluster sweeps to dominance, it is likely to be circulating at a low relative frequency in the range of 1% to 4%. On the negative side, the low surveillance threshold for candidate clusters and consequent lower accuracy require far more surveillance and vaccine development resources than higher surveillance thresholds. In our simulations, for example, the number of clusters screened at the 1% threshold is an order of magnitude higher than at the 10% surveillance threshold and the number of false positive predictions potentially prompting further investigation is also manifold greater.

While our study provides actionable suggestions for improving both the surveillance and forecasting of antigenic turnover, it is limited by several assumptions. One caveat of our method is that we do not capture the explicit phylogenetic structure of the influenza population. Therefore, we do not distinguish between clusters that are successful because of one mutation and clusters that are successful because of a series of mutations. If for instance, a novel antigenic mutation caused the emergence of a new cluster (phenotype) that circulated briefly before a second novel antigenic mutation caused a second phenotype that eventually achieved our defined criteria, we ignore the fact that the established cluster is a subclade of the first and that the antigenic mutation that conferred the first phenotype is fixed along with the second antigenic mutation [34,43]. This scenario follows Koelle & Rasmussen’s description of a two-step antigenic change molecular pathway that leads to antigenic cluster transitions [31]. Our analysis is therefore relevant for scenarios that depict their described jackpot strategy—a combination of one large antigenic mutation occurring on a low deleterious background. Second, the simulation represents global H3N2 dynamics and ignores both differences in temperate and tropical transmission dynamics [44–46] and the emergence of a novel antigenic cluster via importation. Prior studies have revealed considerable global variation in transmission rates, which should positively correlate with the frequency of cluster transitions. Furthermore, viruses that emerge in tropical regions are more likely to be the source of viruses that eventually circulate in temperate regions [47,48]. Temperate regions produce more extreme seasonal bottlenecks, potentially leading to greater stochasticity in viral dynamics, which makes it more difficult for novel strains that emerge in temperate regions to spread globally [49]. For imported viruses, we expect the predictors of success to be similar to those arriving via mutation. However, an importing virus with a large antigenic jump may change the rate of establishment and reduce the time horizon for prediction. We also do not consider selective pressures imposed by seasonal vaccination. Its impact on antigenic turnover depends on vaccination rates and the immunological match between the vaccine and all co-circulating viruses. Seasonal vaccination could differentially modify the effective susceptibility of clusters, suppressing some while creating competitive vacuums for others. Theoretical study suggests that antigenic drift should slow down [50,51] and the circulation of co-dominant clusters may become more common [52]. Given these caveats, we emphasize our qualitative rather than the quantitative results. Our study highlights promising predictors of viral success, characterizes robust trade-offs between the timing, costs and accuracy of such predictions, and serves as proof-of-concept that model-derived surveillance strategies can accelerate and improve forecasts of antigenic sweeps. If we fit similar models to surveillance data as it becomes increasingly available, the resulting predictions will likely reflect greater uncertainty but perhaps naturally reflect global variation in influenza dynamics and vaccination pressures.

Our study demonstrates that the early detection of emerging influenza viruses is limited by a tight race between the typical dynamics of antigenic turnover and the annual timeline for influenza vaccine development. The relatively poor performance of our models on empirical data provides impetus for denser sampling and the development of rapid computational and biological methods for estimating viral fitness. Nonetheless, it provides a foundation for analyzing the costs and benefits of expanding surveillance capacities and shortening the vaccine production pipeline. As we strive to expedite and improve molecular surveillance for vaccine strain selection, even incremental progress is valuable. Earlier detection of antigenic sweeps, regardless of vaccine efficacy, can inform better predictions of severity, public health messaging regarding personal protective measures, and clinical preparedness for seasonal influenza.

## Methods

### Simulation model and data

We implemented a published stochastic individual-based susceptible-infected (SI) phylodynamic model of influenza A/H3N2 [31,32] to repeatedly simulate 30 years of transmission in a well-mixed population of constant size (40 million hosts) with birth and death dynamics (Fig 1A). In brief, each individual host is characterized by its infection status––susceptible or infected––and a history of prior viral infections. Viruses are defined by a discrete antigenic phenotype, which determines the degree of immune escape from other phenotypes, and a deleterious genetic mutation load (*k*) which affects the virus’ transmissibility. Antigenic mutations occur stochastically and confer advanced antigenicity to the virus. We assume that the degree of immune escape conferred by the antigenic mutations follows the gamma distribution described in Koelle & Rasmussen [31] (mean 0.012, shape parameter = 2). New antigenic clusters (phenotypes) are generated when the conferred advanced antigenicity exceeds the mean of this distribution. The probability that a given virus will infect a given host is determined by how similar the antigenic phenotype of the challenging virus is to the antigenic phenotype of the host’s most related previous infection. This probability, or degree of immune escape, is tracked through the simulation by the evolutionary history of clusters (parent-child relationships).

Antigenic and deleterious non-antigenic mutations occur only during transmission events; the model assumes that viruses within a single individual host are genotypically homogeneous. The model also assumes no co-infection, no seasonal forcing [45,53], and no short-term immunity that would broadly prevent reinfection after recovering from infection. We assume parameter values provided in Koelle & Rasmussen [10,31,54–56]. The recovery rate and baseline reproduction number were estimated for influenza A/H3N2, while the evolutionary parameters are based on studies of other RNA viruses including influenza A/H1N1 [10,54–56].

We ran 100 replicate simulations and selected a subset that produced realistic global influenza dynamics. Specifically, we excluded 38 simulations in which endemic transmission died out prior to the 30 years. We treated the first five years of each simulation as burn-in periods. In total, we analyzed 1550 years of simulated influenza transmission and evolutionary dynamics.

Throughout each simulation, we tracked 23 metrics reflecting the epidemiological state of the host population (i.e., number of susceptible and infected individuals) and evolutionary state of the viral population (S2 Table) at 14-day intervals. When possible, we monitored these quantities for both individual antigenic clusters and the entire viral population, and then calculated their ratio. For example, we monitored the average number of deleterious mutations within each antigenic cluster and across all viruses, as well as the *relative* mutational load of each cluster with respect to the entire viral population. Henceforth, we refer to the metrics as *candidate predictors*.

We classified each novel antigenic cluster in each simulation into one of three categories: (1) rapidly eliminated clusters that never reach 1% relative frequency in the population, (2) transient clusters that surpass 1% relative frequency but do not qualify as established clusters, and (3) established clusters that circulate above 20% relative frequency for at least 45 days. With this criteria, transient and established clusters constituted on average 81% of the infections at any point in time (S1 and S2 Figs).

### Predictive models

Restricting our analysis to transient and established clusters, we used generalized linear modeling to identify important early predictors of evolutionary fate. For each antigenic cluster, we predicted its evolutionary future (i.e., whether it ultimately becomes established) at specified surveillance thresholds, such as 5% relative frequency. Specifically, we recorded all candidate epidemiological and evolutionary predictors at the moment each cluster crossed the threshold. We analyzed all ten surveillance thresholds ranging from 1% to 10% at 1% increments. When a rising cluster reached 1% relative frequency, the median prevalence of infections caused by the cluster was 118 (IQR: 65–205); at 10% relative frequency, the median prevalence was 1039 (IQR: 503–1940).

For each surveillance threshold, we centered and scaled candidate predictors and removed collinear factors. Using five-fold cross validation, we partitioned the data into five subsets, keeping data from individual simulations in the same subsets. We fit mixed-effects logistic regression models using four subsets for training and controlling for differences between independent simulations. Predictors were added sequentially based on which term lowered the average Akaike Information Criterion of the five training folds the most and provided a statistically significant better fit than the reduced model.

We evaluated model performance by predicting the evolutionary outcomes of clusters in the held-out test subset. We calculated three metrics: the area under the receiver operating curve (AUC), the sensitivity (the proportion of all positives predicted as positive), and the positive predictive value (the proportion of true positives of all predicted positives). The model predicts the probability that a cluster will establish. To translate these outputs into discrete binary predictions of future success, we applied a probability threshold which maximized the F1 score [57], which is the harmonic mean of a model’s positive predictive value and sensitivity (S2 Table). When we included historical data of candidate predictors, i.e. the value of a candidate predictor at an earlier surveillance threshold, positive predictive values were marginally higher, while sensitivity values were similar to those from models only the current surveillance threshold data (S5 Fig).

We also considered an opportunistic sampling regime, where samples are tested as they arise regardless of their relative frequency. We fit models aimed at two prediction targets: (1) the evolutionary success of a cluster sampled at an arbitrary relative frequency and (2) the frequency of a cluster up to twelve months into the future. We built models based on data sampled from ten random time points in each of the 62 25-year simulations. We considered all clusters present above 1% relative frequency but not yet established as a dominant cluster. The frequency of a cluster at the time of sampling was included as an additional predictor. To predict the frequency of an antigenic cluster *X* months into the future, we fit a two-part model that first predicted whether the cluster would be present at the specified date, and, if so, then estimated the frequency of the cluster at that date. We used forward variable selection and cross validation model, as described above. We used the R statistical language version 3.3.2 [58] for all analyses, and the *afex* package for generalized linear models [59].

### Candidate predictors

#### Reproductive rates

In our simulated data, we can calculate the instantaneous reproductive rate for particular clusters and the entire viral population. As described in Koelle & Rasmussen [31], the reproductive rate of a virus *v* is given by:

$$R(v)=\frac{{\beta_{0}(1-s_{d})}^{k(v)}}{\mu +v}\frac{S_{eff}(v)}{N},$$ (1)

Where *β*_0_ is the inherent transmissibility, *s*_*d*_ is the fitness effect for each of the virus’ *k*(*v*) deleterious mutations, *μ* and *v* are the per capita daily death and recovery rates, respectively, and *N* is the host population size. We assume that *β*_0_, *s*_*d*_, *μ* and *v* are constant across all viruses. *S*_*eff*_(*v*) denotes the population-wide susceptibility to the virus accounting for cross-immunity from prior infections, herein referred to as the effective susceptibility, and the population level effective susceptibility is estimated for a virus as:

$$S_{eff}(v)=\frac{S}{N}\sum_{h=1}^{N}\sigma_{v}(h)$$ (2)

where *σ*_*v*_(*h*) is the immunity of host *h* towards virus *v* based on the antigenic similarity between *v* and the virus in host *h*’s infection history most antigenically similar to virus *v*. A *σ*_*v*_(*h*) = 1 indicates full susceptibility, while *σ*_*v*_(*h*) = 0 indicates complete immunity.

The growth rate of an antigenic cluster is then the average *R* over all viruses in that cluster, given by

$$R_{c}=\frac{1}{I_{c}}\sum_{i=1}^{I_{c}}R(v_{i})$$ (3)

where *I*_*c*_ is the number of hosts infected by a virus from cluster c and *v*_*i*_ is the virus infecting host *i*. Likewise the population-wide average (〈*R*〉) and variance (var(*R*)) in *R* are computed across all current infections, and the relative reproductive rate of a cluster is given by *R*_*c*_/〈*R*〉.

#### Practical approximations on simulated data

Eqs (1–3) are not easily calculated from current surveillance data. Therefore, we considered two proxy measures of viral growth rates and two proxy measures of viral competition. We first choose two surveillance thresholds, for example, 6% and 10%. When the relatively frequency of a cluster crosses the second threshold, we calculate both the *time elapsed* since it crossed the first threshold and the *relative fold change*, as given by

$$\chi_{c}\left(t_{1},\;t_{2}\right)=\frac{\Delta_{c}\left(t_{1},t_{2}\right)}{\frac{1}{N_{c}}\sum_{j=1}^{N_{c}}\Delta_{j}\left(t_{1},t_{2}\right)},$$ (4)

Where *t*_1_ and *t*_2_ are the times at which cluster c crossed the first and second threshold, respectively, Δ_*c*_(*s*,*t*) is its relative frequency at time *t* divided by its relative frequency at time *s* and *N*_*c*_ is the number of distinct clusters present at both time *t*_1_ and *t*_2_. For the competition proxy measures, we calculate the variance in *χ*_*c*_(*t*_1_,*t*_2_) and the *N*_*c*_ where Δ_*c*_(*s*,*t*)>1.

We evaluate the performance of these approximations by comparing logistic regression models that predict whether a cluster will establish from either the true *R*_*c*_/〈*R*〉 at the 10% surveillance threshold, the relative fold change between the 6% and 10%, or time elapsed between reaching the 6% and 10% thresholds. As before, we evaluated model performance based on AUC, positive predictive value, and sensitivity.

### Proxy modeling using global influenza surveillance data

We developed proxy models to predict the cluster success of 6271 geographically diverse influenza A/H3N2 sequences sampled from 2006–2018. Clusters were distinguished by single mutations in epitope sites on the HA1 sequence. All strains within a cluster have the same number of epitope mutations with respect to a reference strain, which is defined as having zero epitope mutations. Thus, clusters in the empirical data are defined by the number of epitope mutations. This is in comparison to clusters in the simulated data, which are defined by their degree of immune escape conferred by antigenic mutations. To estimate frequency in the population, we calculated the number of sequences belonging to a cluster over all sequences sampled in a two-month moving window. Using the estimated frequency and the number of epitope mutations in a cluster, we derived twenty measures of fitness and competition based on the first two time points a cluster was sampled (S6 Table). Only 378 of the 1516 unique clusters sampled from 2006–2018 were sampled at least twice and thus used to fit the models. We tested all combinations of fitness and competition measures (N = 121) in a logistic regression framework, using the AUC, positive predictive value, and sensitivity as performance measures (S7 Table). All scripts and data files to recreate the analysis are provided within the S1 Source file.

## Supporting information

S1 Fig The proportion of total infections caused by established clusters is more sensitive to a frequency criterion than the duration of time. Established antigenic clusters account for the majority of the disease activity. In our analysis, established clusters are those that circulate above 20% relative frequency for at least 45 days. We choose the most stringent criteria that, when only accounting for clusters that reached the criteria, still maintained the overall cyclical influenza dynamics.
(TIF)

S2 Fig Distinguishing cluster behavior. (a) Histograms of the number of days established and transient clusters were above 20% relative frequency. The red line designates the 45-day threshold we used in criteria for defining successful clusters. (b) Established clusters circulate longer at higher frequencies than transient clusters. Considering a cluster's maximum relative frequency in the population and how many days it circulated above 20% relative frequency, two groups of clusters emerge around the one to two-month mark (dashed lines). We do not suspect our results would be sensitive to slight changes in the day criterion within this range as the number of clusters in this range represent less than 1% of our sample. We chose 45 days as a balance between confirming the cluster circulated at a sufficient level to possibly warrant public health attention and reducing false positives
(TIF)

S3 Fig The fate of novel antigenic clusters. (a) Each point represents the number of antigenic clusters in our simulations that reach increasing surveillance thresholds (i.e., relative frequency in the population). As the surveillance threshold increases from 1 to 10%, the number of candidate clusters decreases from 7969 clusters at the 1% threshold to 1816 clusters at the 10% surveillance threshold. Each point represents the number of data points we used to construct the predictive models for a given surveillance threshold. (b) Given a cluster has reached a surveillance threshold, the proportion of antigenic clusters that will establish (i.e. reaches > 20% for 45 days) increases with higher surveillance thresholds.
(TIF)

S4 Fig **The rate of change (a) and relative fold change (b) as proxy measures for the relative growth rate *R***_***c***_**/〈*R*〉.** Because the top selected predictors across all models cannot easily be estimated using readily available surveillance data, we evaluated several proxy measures of viral growth rates and viral competition. We compared approximations that measure growth rates. Contour lines indicate the density of values for clusters that establish (black, N = 1126) and those that transiently circulate (grey, N = 723). Values along the x-axis indicate the empirical relative growth rate of a cluster the moment it reaches the 10% surveillance threshold. Values along the y-axis indicate the proxy measure (rate of change in (a) and relative fold change in (b) for the cluster, approximated for the time between the 6% and 10% surveillance thresholds.) The rate of change, measured in the number of days between the two thresholds, is a better proxy measure than relative fold change.
(TIF)

S5 Fig Predictive models that rely on data from a single sampling event perform similarly to those that include data from multiple sampling events. Each point represents the performance of the optimal combination of candidate predictors that best predicts the evolutionary fate of antigenic clusters under two different surveillance strategies, whether the model includes data from a single time point (yellow) or multiple time points (purple). For a target surveillance threshold, models that incorporated data from multiple sampling events used data from the 1% surveillance threshold, the mid-point relative frequency, and the target surveillance threshold. For the multiple sampling event models, candidate predictors included all variables listed in S1 Table, as well as the difference in predictor values between 1% and the mid-point relative frequency, and the mid-point relative frequency and the target surveillance threshold. Dots represent the median, and vertical error bars span the range of performance values across the five folds of cross-validation of the best-fit model. In our main results we focus on strategy that only incorporates current data because of the simplicity in the methodology and reduction of candidate predictors.
(TIF)

S6 Fig Frequency distribution of 2846 clusters from 620 random time samples across the 1500 years of simulated seasonal influenza dynamics. In addition to a surveillance threshold sampling regime, we considered opportunistic sampling. Clusters that were below 1% relative frequency in the population or those that had already reached our establish criteria were excluded. Because the majority of clusters are sampled at low relative frequencies, predictive models built on an opportunistic sampling scheme performed similarly to predictive models at low surveillance thresholds.
(TIF)

S7 Fig The sensitivity and positive predictive value trade-off of two surveillance strategies: 1) surveillance threshold (circles, triangles) and 2) random sampling through time (squares). Each set of symbols highlights the tradeoff between sensitivity and positive predictive value at different probability thresholds for what constitutes a positive prediction, i.e. a future successful cluster. All models converge in areas with low sensitivity and high positive predictive value, where the probability threshold for what classifies as a positive prediction is 0.9. However, in regions of greater sensitivity, the random sample time model consistently underperforms models that use a < 5% surveillance threshold. The black stars represent the probability threshold that maximizes the F1 value, the harmonic average of a model's positive predictive value and sensitivity.
(TIF)

S8 Fig Three-month incremental test model predictions of cluster frequency up to a year in advance. To predict the frequency of an antigenic cluster in X months in the future, we fit a two-part model that first predicted whether the cluster would be present at the specified date, and if so, then the estimated frequency. The number of clusters present at the time of initial sampling, but expected to persist in the future, decreases with increasing month-ahead predictions. Out of 2846 unique clusters, 2279 cluster were present above 1% relative frequency at 3 months; 1921 clusters at 6 months, 1624 clusters at 9 months, and 1378 clusters at 12 months. As the models predict further into the future, the model underestimates future-high frequency circulating clusters, which are usually clusters that will establish. We tested the performance of the best-fit model for each 3-month increment on a new data set consisting of 5 random time points over a 25-year period, corresponding to 310 time points over all 62 simulations. These test predictions are shown in panels a-d. To improve model fit, the target frequency, f_c_' in X months was log-transformed. The black line represents perfect agreement between the actual and predicted log frequencies. Black dots represent clusters that will eventually establish, and grey dots are clusters that will transiently circulate. In addition, we tried fitting the model the frequency fold in X months’ time, i.e. f_c(t+x)_ /f_c(t)_; however, the model's goodness-of-fit, as measured by the adjusted R^2^ was consistently lower than that of the models predicting the log-transformed frequency.
(TIF)

S1 Table Full set of candidate predictors considered. Values were taken at the moment a focal antigenic cluster reached a specified surveillance threshold. The columns *Population*, *Cluster*, *Relative* indicate the scale and measure (e.g. mean and/or variance) that a predictor was considered in the model. Depending on the scale of the predictor, the *formula* could refer to all strains in the population, i.e. the strains of infected hosts, or the subset of strains in a specific cluster. *For computational simplicity, these quantities were calculated using strains from a random sample of 10,000 infected individuals. N = number of hosts; *t*_*a0*_ = the time of birth of virus a; *λ* = antigenic distance between two strains. The antigenic distance is the pairwise degree of cross-immunity between two strains determined by the size of antigenic mutations and parent-offspring relationships; *k(v*_*i*_*)* = the number of deleterious mutations on a virus *v* of infected host *i*; *s*_*d*_ = the fitness effect of a deleterious mutation; *σ*_*v*_ = the average individual population susceptibility to cluster *c*; *σ*_*v*,*c*(*h*,*v*)_ = the probability of infection of a host with historical infection *i* by a strain of cluster *v*.
(PDF)

S2 Table Best-fit model results for surveillance thresholds 1–10%. The predictor variables are listed in the order by which they were selected using a forward selection algorithm. The coefficient estimate is the maximum and minimum coefficient (log-odds) from the five-fold cross validation of the final full-term model with the corresponding std. error.
(PDF)

S3 Table Evaluating proxy measures for different phases of a novel antigenic cluster's early expansion. Model 1 shows the performance of the best-fit model using the actual values of relative fitness (relative growth rate) and competition (variance in the population growth rate) for clusters that reached the 5% surveillance thresholds (top two sections) and the 10% surveillance threshold (bottom two sections). Within each section, Model 2 substituted a time proxy for the fitness term and the absolute number of clusters that were growing for the competition term. Model 3 substituted a relative fold change for the fitness term and the population-wide variance in fold change for the competition term. *t*_*1*_ is when a focal cluster reaches the lower surveillance threshold (1%, 3%, 6%, 8%); *t*_*2*_ is when the same cluster reaches the higher surveillance threshold (5%, 10%) Performance metric values are the median across the five folds in cross-validation. Balanced accuracy measures the accuracy of the model, accounting for the imbalance in outcomes (i.e. number of transient versus established clusters) in the data set. In addition, to the terms included in the table, we tested the fold change of the dominant cluster from *t*_*1*_ and *t*_*2*_ as a predictor, but did not find that this term was a significant proxy in any model.
(PDF)

S4 Table Best-fit logistic regression results for predicting presence-absence of a cluster in X months’ time into the future. Terms are listed in the order they were added to the model through forward-selection.
(PDF)

S5 Table Best-fit linear regression models for predicting frequency of a cluster in X months’ time into the future. Terms are listed in the order they were added to the model through forward-selection. The Adjusted R^2^ and Root Mean Squared Error (RMSE) were measured on a testing data set of 5 random time samples over a 25-year period.
(PDF)

S6 Table Full set of candidate predictors considered for empirical models. Values were taken at the first two sample points a focal antigenic cluster was recorded. All viruses within a cluster have the same number of epitope mutations, *ρ*. Symbols for quantities are consistent with S1 and S3 Tables.
(PDF)

S7 Table Top 10 Performing Empirical Models ranked by F1 score. *All models marked have the same F1 score and are listed in order of descending AUC score. The AUC is the average of the AUC from the testing data, while the PPV and Sensitivity are measured at the threshold that maximizes the F1 score. For all empirical models, the frequency of the focal cluster, *f*_c_, at either *t*_1_ or *t*_2_ was the best fitness measure. The competition term either captured the average or variance in the frequency of competing clusters or how the absolute number of competing changes changed from *t*_1_ to *t*_2_.
(PDF)

S1 Source R source code and csv data files for model fitting and figure generation. (GZ)

S1 File Acknowledgments for all of the sequences used in the empirical analysis. (XLSX)
