# Supplementary figures and images for: Early prediction of antigenic transitions for influenza A/H3N2
Source: PLoS Comput Biol. 2020 Feb 18;16(2):e1007683. doi: 10.1371/journal.pcbi.1007683 (PMC7048310; doi:10.1371/journal.pcbi.1007683)

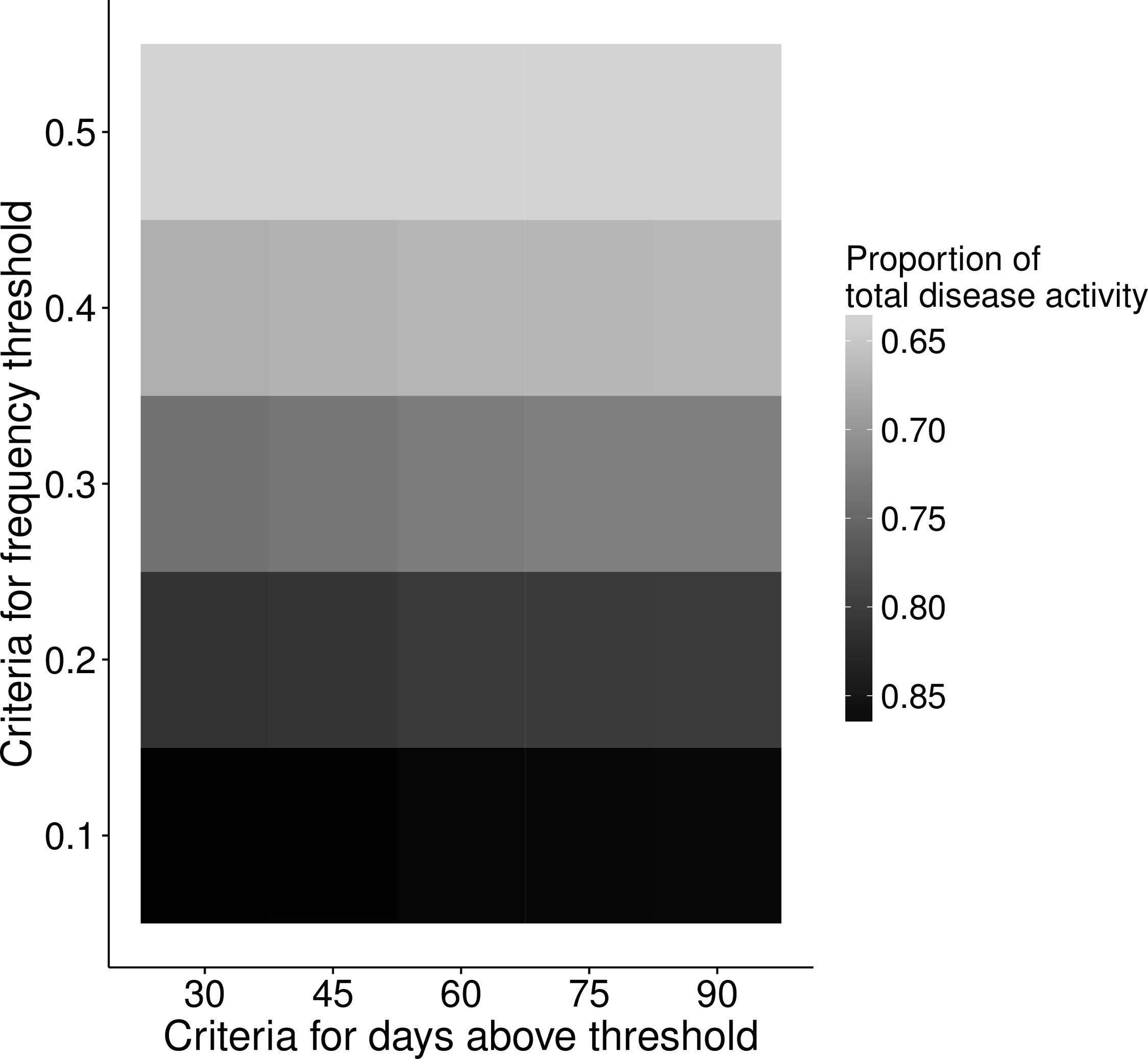

Supplement: S1 Fig — Established antigenic clusters account for the majority of the disease activity. In our analysis, established clusters are those that circulate above 20% relative frequency for at least 45 days. We choose the most stringent criteria that, when only accounting for clusters that reached the criteria, still maintained the overall cyclical influenza dynamics. (TIF) [file pcbi.1007683.s001.tif]

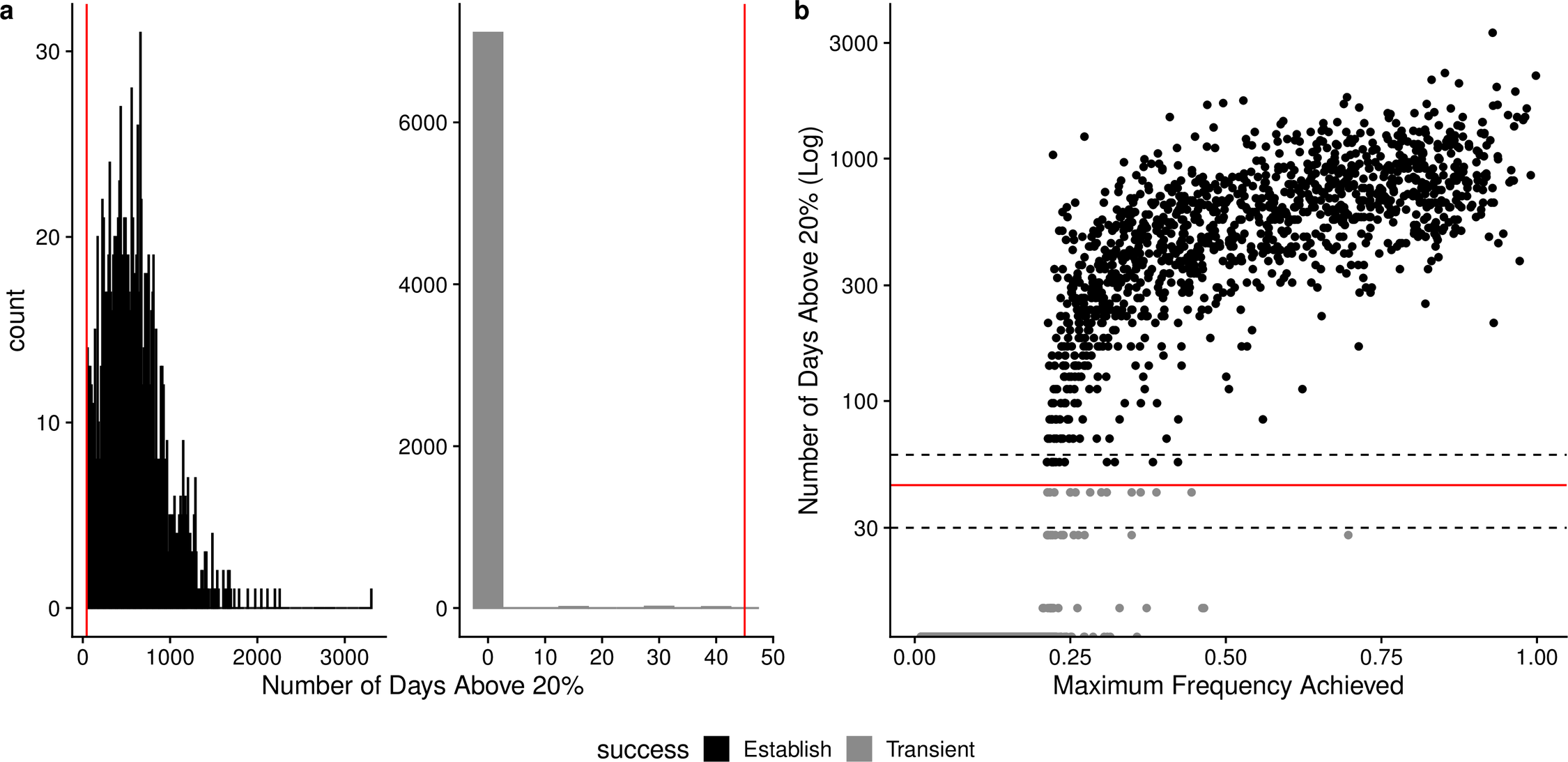

Supplement: S2 Fig — (a) Histograms of the number of days established and transient clusters were above 20% relative frequency. The red line designates the 45-day threshold we used in criteria for defining successful clusters. (b) Established clusters circulate longer at higher frequencies than transient clusters. Considering a cluster's maximum relative frequency in the population and how many days it circulated above 20% relative frequency, two groups of clusters emerge around the one to two-month mark (dashed lines). We do not suspect our results would be sensitive to slight changes in the day criterion within this range as the number of clusters in this range represent less than 1% of our sample. We chose 45 days as a balance between confirming the cluster circulated at a sufficient level to possibly warrant public health attention and reducing false positives (TIF) [file pcbi.1007683.s002.tif]

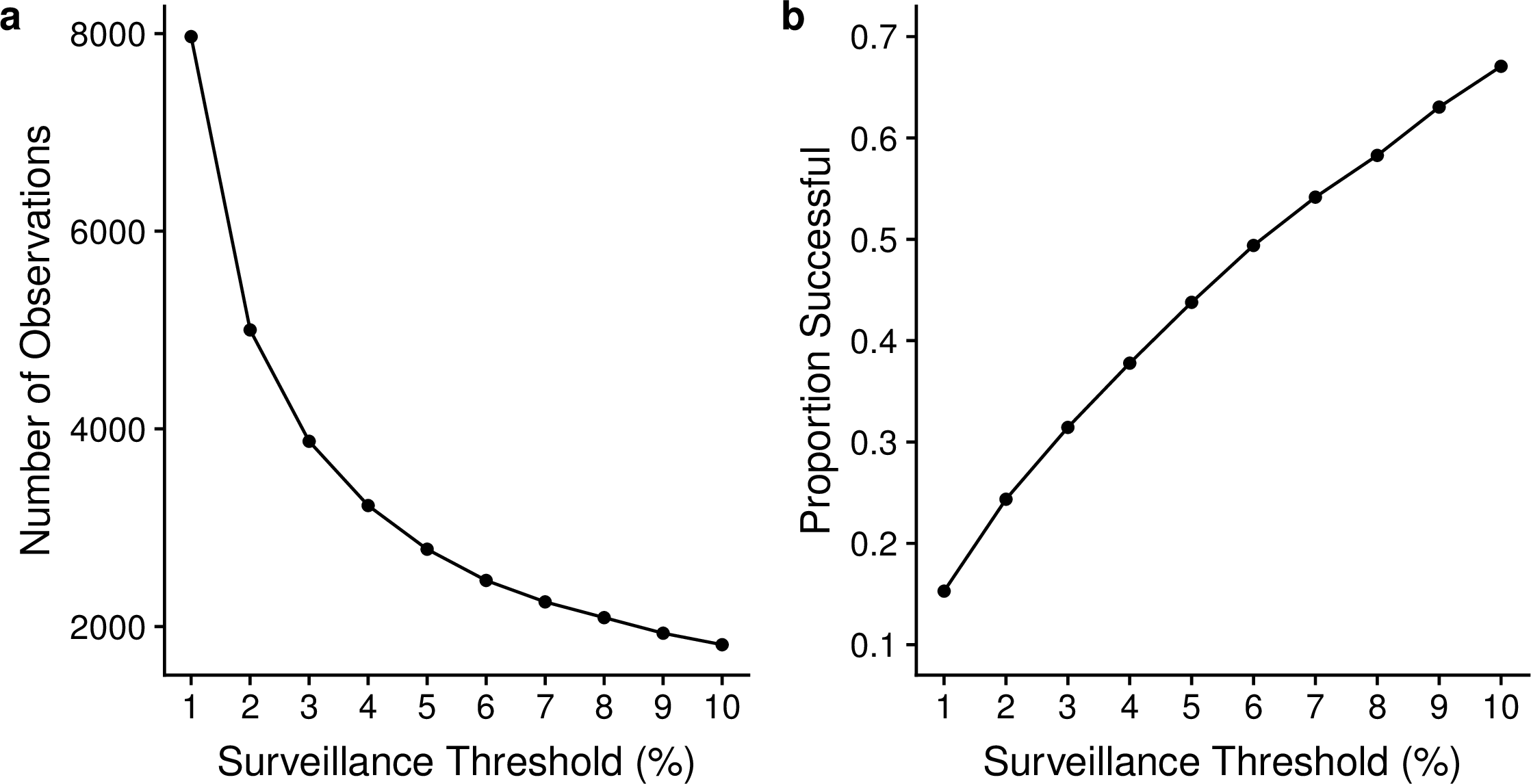

Supplement: S3 Fig — (a) Each point represents the number of antigenic clusters in our simulations that reach increasing surveillance thresholds (i.e., relative frequency in the population). As the surveillance threshold increases from 1 to 10%, the number of candidate clusters decreases from 7969 clusters at the 1% threshold to 1816 clusters at the 10% surveillance threshold. Each point represents the number of data points we used to construct the predictive models for a given surveillance threshold. (b) Given a cluster has reached a surveillance threshold, the proportion of antigenic clusters that will establish (i.e. reaches > 20% for 45 days) increases with higher surveillance thresholds. (TIF) [file pcbi.1007683.s003.tif]

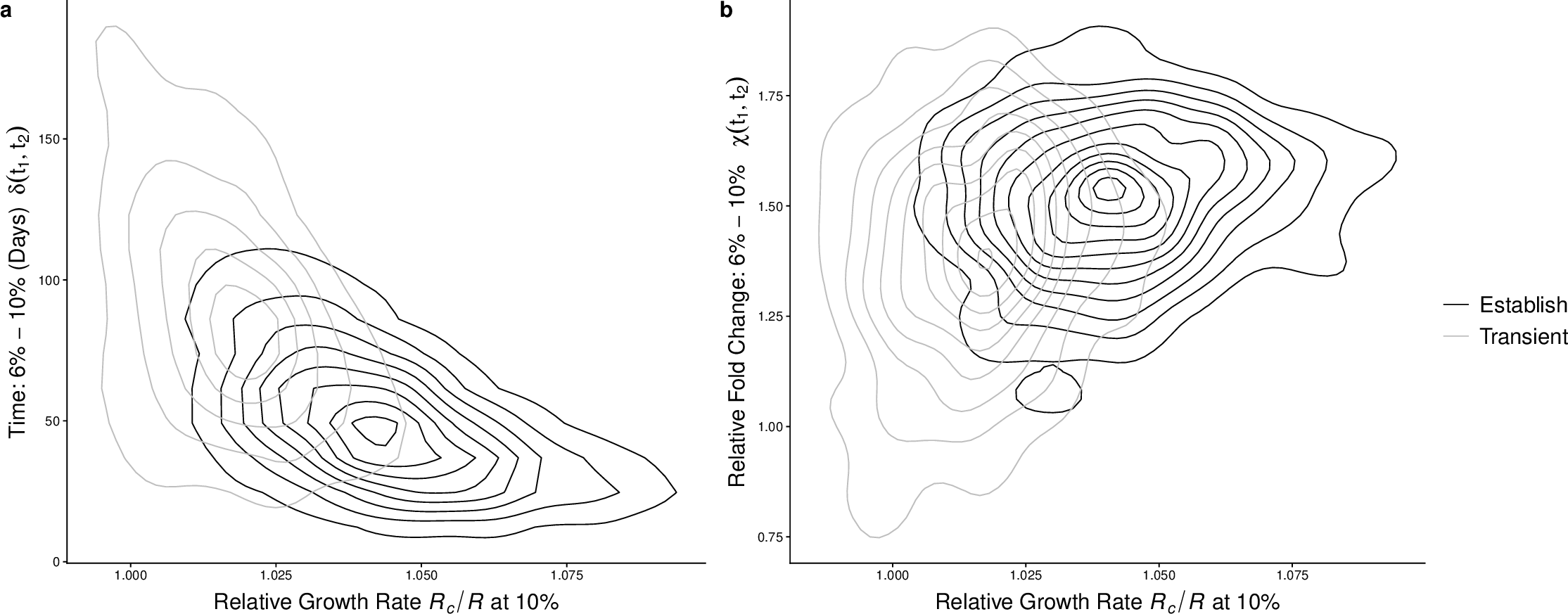

Supplement: S4 Fig — The rate of change (a) and relative fold change (b) as proxy measures for the relative growth rate Rc/〈R〉. Because the top selected predictors across all models cannot easily be estimated using readily available surveillance data, we evaluated several proxy measures of viral growth rates and viral competition. We compared approximations that measure growth rates. Contour lines indicate the density of values for clusters that establish (black, N = 1126) and those that transiently circulate (grey, N = 723). Values along the x-axis indicate the empirical relative growth rate of a cluster the moment it reaches the 10% surveillance threshold. Values along the y-axis indicate the proxy measure (rate of change in (a) and relative fold change in (b) for the cluster, approximated for the time between the 6% and 10% surveillance thresholds.) The rate of change, measured in the number of days between the two thresholds, is a better proxy measure than relative fold change. (TIF) [file pcbi.1007683.s004.tif]

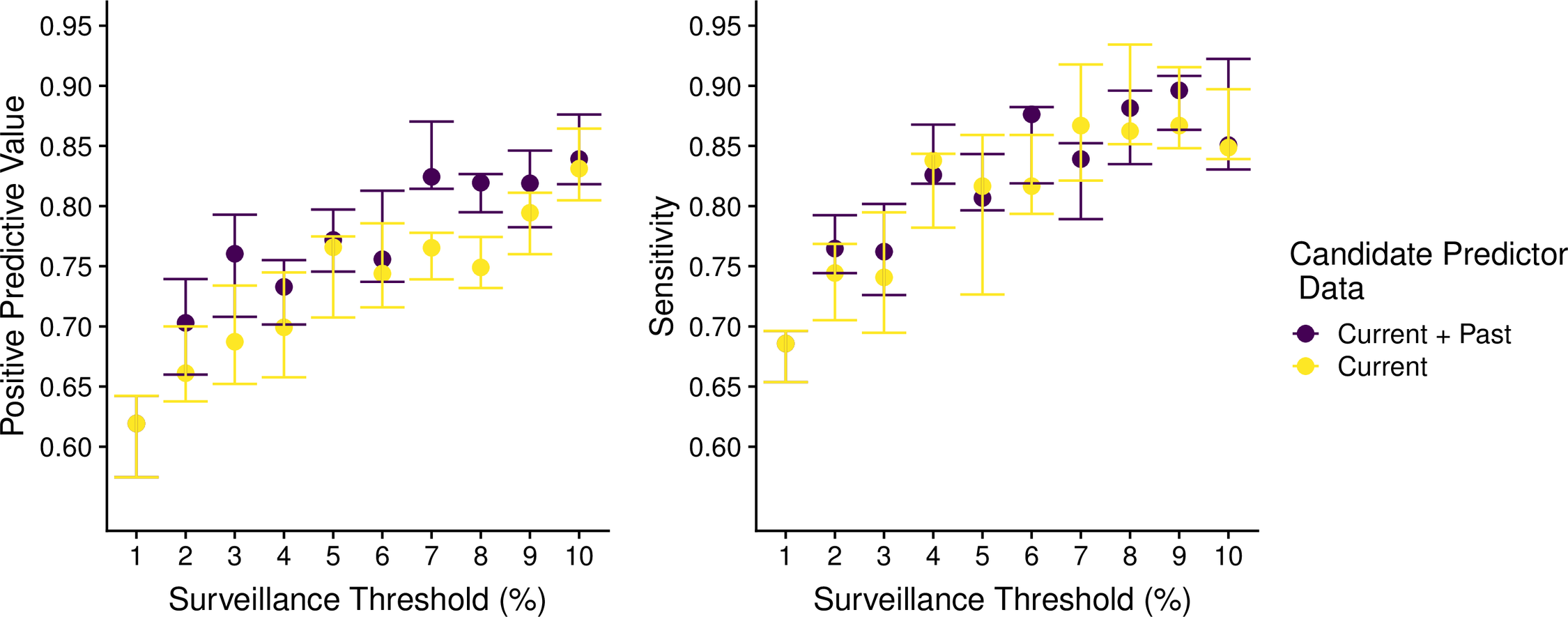

Supplement: S5 Fig — Each point represents the performance of the optimal combination of candidate predictors that best predicts the evolutionary fate of antigenic clusters under two different surveillance strategies, whether the model includes data from a single time point (yellow) or multiple time points (purple). For a target surveillance threshold, models that incorporated data from multiple sampling events used data from the 1% surveillance threshold, the mid-point relative frequency, and the target surveillance threshold. For the multiple sampling event models, candidate predictors included all variables listed in S1 Table, as well as the difference in predictor values between 1% and the mid-point relative frequency, and the mid-point relative frequency and the target surveillance threshold. Dots represent the median, and vertical error bars span the range of performance values across the five folds of cross-validation of the best-fit model. In our main results we focus on strategy that only incorporates current data because of the simplicity in the methodology and reduction of candidate predictors. (TIF) [file pcbi.1007683.s005.tif]

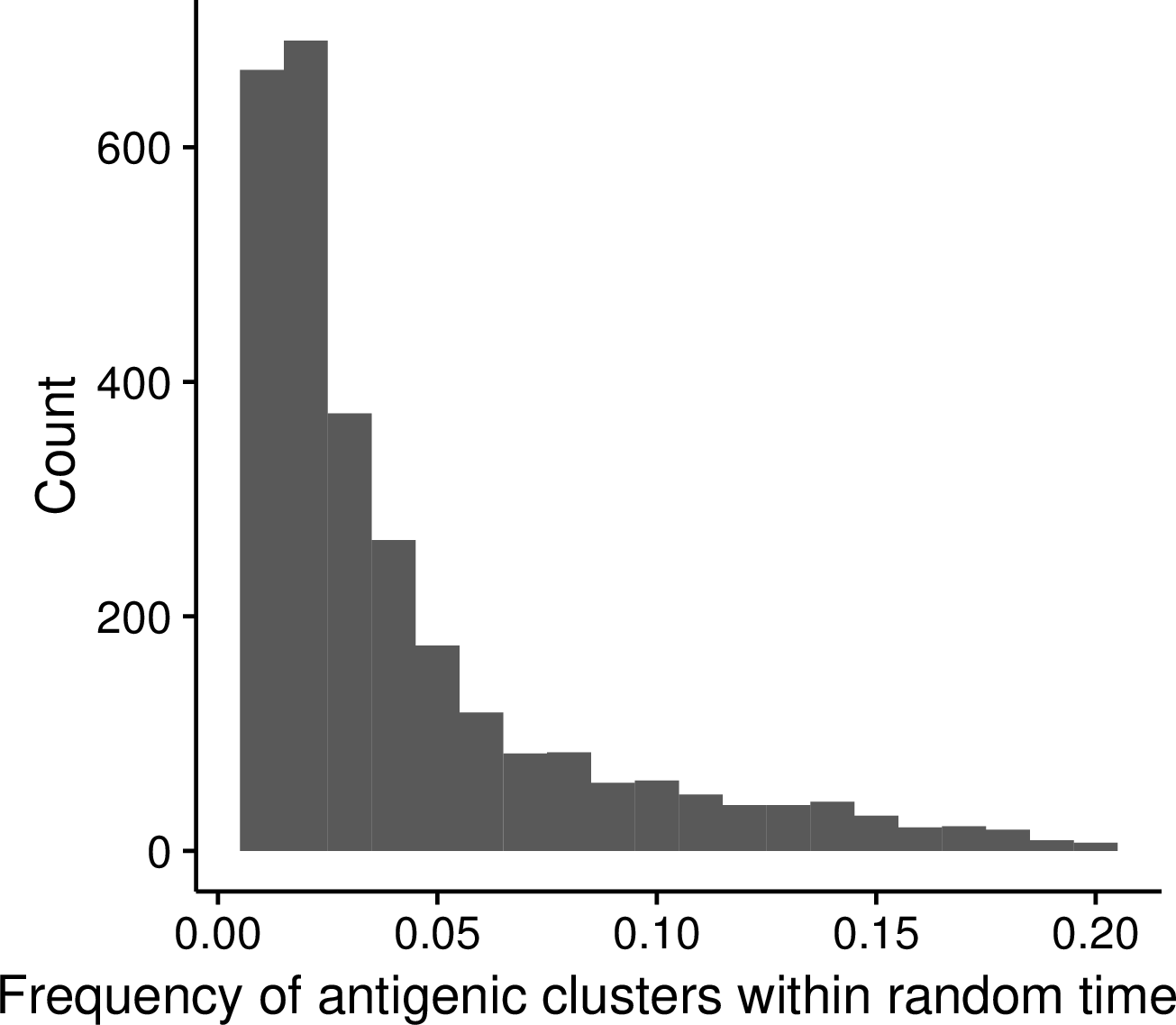

Supplement: S6 Fig — In addition to a surveillance threshold sampling regime, we considered opportunistic sampling. Clusters that were below 1% relative frequency in the population or those that had already reached our establish criteria were excluded. Because the majority of clusters are sampled at low relative frequencies, predictive models built on an opportunistic sampling scheme performed similarly to predictive models at low surveillance thresholds. (TIF) [file pcbi.1007683.s006.tif]

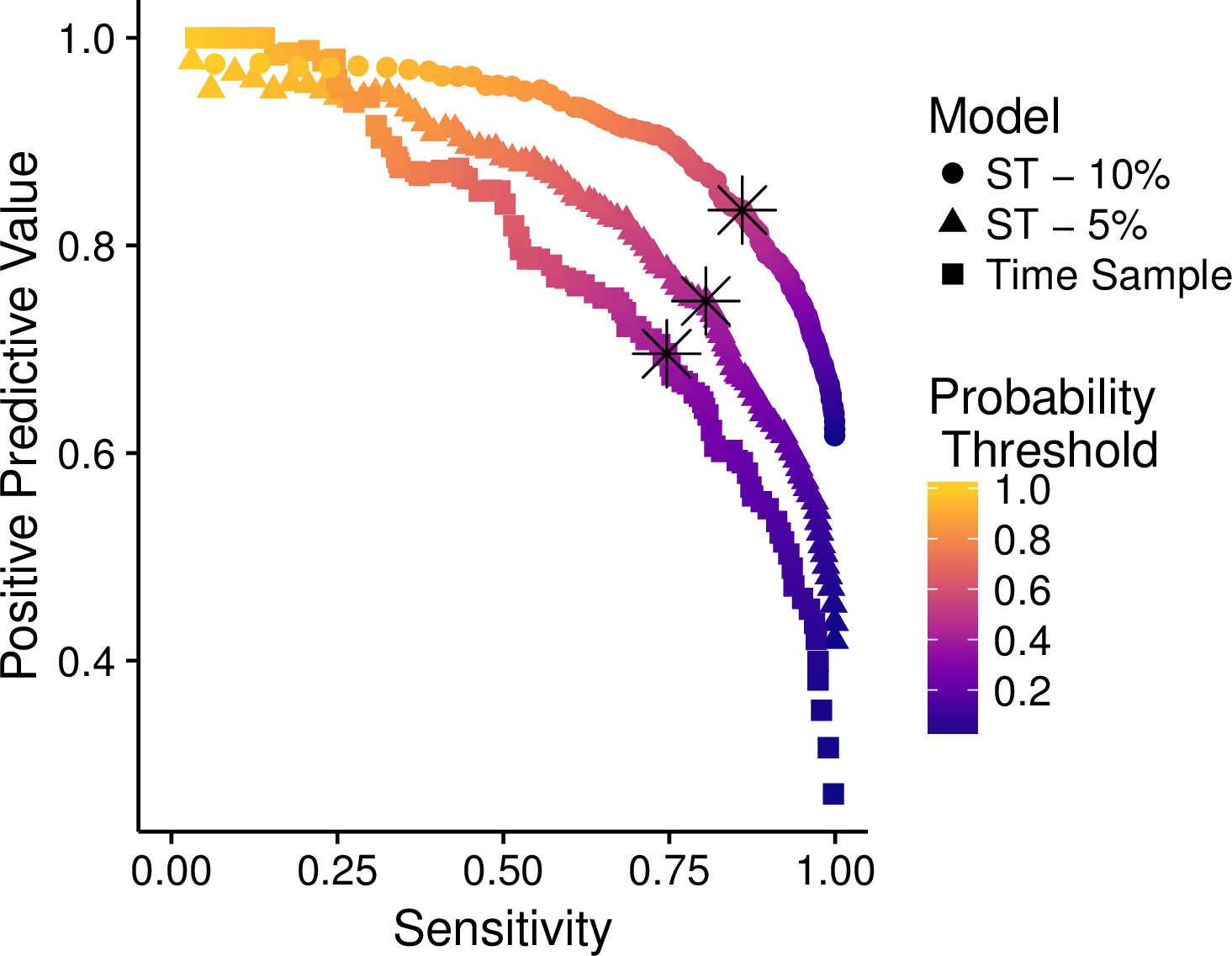

Supplement: S7 Fig — Each set of symbols highlights the tradeoff between sensitivity and positive predictive value at different probability thresholds for what constitutes a positive prediction, i.e. a future successful cluster. All models converge in areas with low sensitivity and high positive predictive value, where the probability threshold for what classifies as a positive prediction is 0.9. However, in regions of greater sensitivity, the random sample time model consistently underperforms models that use a < 5% surveillance threshold. The black stars represent the probability threshold that maximizes the F1 value, the harmonic average of a model's positive predictive value and sensitivity. (TIF) [file pcbi.1007683.s007.tif]

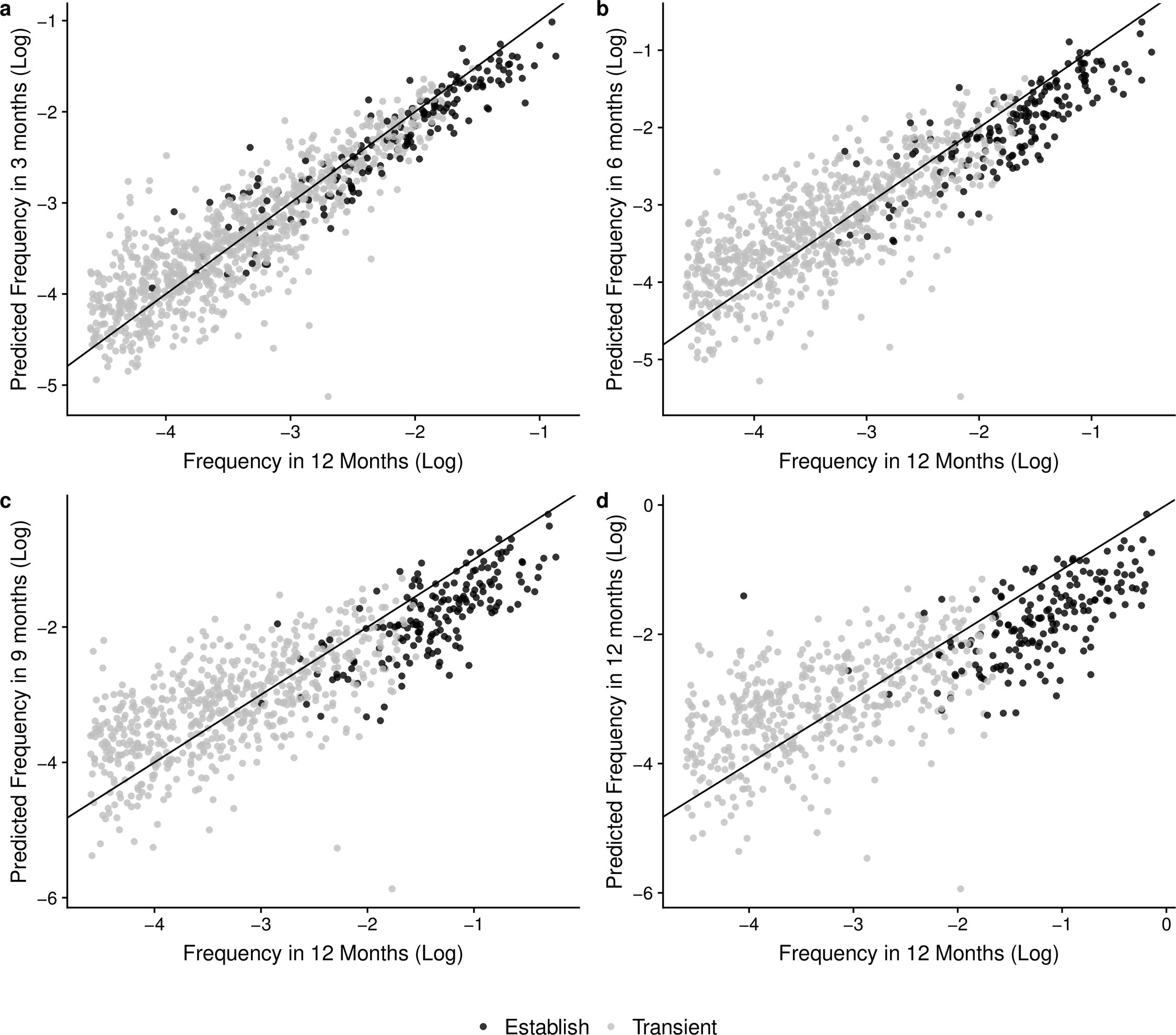

Supplement: S8 Fig — To predict the frequency of an antigenic cluster in X months in the future, we fit a two-part model that first predicted whether the cluster would be present at the specified date, and if so, then the estimated frequency. The number of clusters present at the time of initial sampling, but expected to persist in the future, decreases with increasing month-ahead predictions. Out of 2846 unique clusters, 2279 cluster were present above 1% relative frequency at 3 months; 1921 clusters at 6 months, 1624 clusters at 9 months, and 1378 clusters at 12 months. As the models predict further into the future, the model underestimates future-high frequency circulating clusters, which are usually clusters that will establish. We tested the performance of the best-fit model for each 3-month increment on a new data set consisting of 5 random time points over a 25-year period, corresponding to 310 time points over all 62 simulations. These test predictions are shown in panels a-d. To improve model fit, the target frequency, fc' in X months was log-transformed. The black line represents perfect agreement between the actual and predicted log frequencies. Black dots represent clusters that will eventually establish, and grey dots are clusters that will transiently circulate. In addition, we tried fitting the model the frequency fold in X months’ time, i.e. fc(t+x) /fc(t); however, the model's goodness-of-fit, as measured by the adjusted R2 was consistently lower than that of the models predicting the log-transformed frequency. (TIF) [file pcbi.1007683.s008.tif]
