# Supplementary material for: Early prediction of antigenic transitions for influenza A/H3N2
Source: PLoS Comput Biol. 2020 Feb 18;16(2):e1007683. doi: 10.1371/journal.pcbi.1007683 (PMC7048310; doi:10.1371/journal.pcbi.1007683)
Supplement: S1 Table — Values were taken at the moment a focal antigenic cluster reached a specified surveillance threshold. The columns Population, Cluster, Relative indicate the scale and measure (e.g. mean and/or variance) that a predictor was considered in the model. Depending on the scale of the predictor, the formula could refer to all strains in the population, i.e. the strains of infected hosts, or the subset of strains in a specific cluster. *For computational simplicity, these quantities were calculated using strains from a random sample of 10,000 infected individuals. N = number of hosts; ta0 = the time of birth of virus a; λ = antigenic distance between two strains. The antigenic distance is the pairwise degree of cross-immunity between two strains determined by the size of antigenic mutations and parent-offspring relationships; k(vi) = the number of deleterious mutations on a virus v of infected host i; sd = the fitness effect of a deleterious mutation; σv = the average individual population susceptibility to cluster c; σv,c(h,v) = the probability of infection of a host with historical infection i by a strain of cluster v. (PDF) [file pcbi.1007683.s009.pdf]

Candidate Predictor	Formula	Population	Cluster	Relative
Number of Infected Individuals	I	X		
Number of Uninfected Individuals	S	X		
Proportion of Individuals Infected	I/N	X		
Number of Circulating Antigenic Clusters	N_{c}	X		
Frequency of Current Dominant Cluster	$f_c = \max\left[I_c/I\right]$	X		
Entropy (Shannon's Diversity Index)	$H = \frac{1}{N_C}\sum_{j=1}^{N_C} f_j \ln\frac{1}{f_j}$	X		
Serial Interval of Infection*	$\mathrm{SI} = \frac{1}{I}\sum_{a,b\in\mathrm{infecteds}}(t_{a_0} - t_{b_0})$	X		
The most recent common ancestor*	$\mathrm{TMRCA} = \max\left[\frac{1}{2}\left(\left(t_{TMRCA_0} - t_{a_0}\right) + \left(t_{TMRCA_0} - t_{b_0}\right)\right)\right]$	X		
Genetic Diversity*	$\omega = \frac{1}{I}\sum_{a,b\in\mathrm{infecteds}}\frac{1}{2}\left(\left(t_{TMRCA_0} - t_{a_0}\right) + \left(t_{TMRCA_0} - t_{b_0}\right)\right)$	X		
Antigenic Diversity*	$\lambda = \frac{1}{I}\sum_{a,b\in\mathrm{infecteds}}\lambda_{ab}$	X		
Deleterious Mutational Load	$\mathrm{k} = \frac{1}{I}\sum_{i=1}^{I} k(v_i)$	Mean, Var	Mean, Var	Mean, Var
Transmissibility	$\beta = \frac{1}{I}\sum_{i=1}^{I}\beta_0\left(1 - s_d\right)^{k(v_i)}$	Mean, Var	Mean, Var	Mean, Var
Effective Susceptibility*	$S_{\mathrm{eff}}(v) = \frac{S}{N}\sum_{h=1}^{N}\left(\sigma_{v(h)}\right)$	Mean, Var		
Covariance in transmissibility and effective susceptibility	$\mathrm{cov} = \frac{1}{I}\sum_{i=1}^{I}\left((\beta_i - \bar{\beta}) * (\sigma_v(i) - \bar{\sigma})\right)$	X		
Cluster Susceptibility*	$\sigma(v) = \sum_{h=1}^{N}\min(1, \sigma_{v,c(h,v)})$		Mean, Var	
Reproductive Growth Rate	$R(v) = \frac{\beta_0\left(1-s_d\right)^{k(v)}}{\mu+\nu}\left(\frac{S_{\mathrm{eff}}(v)}{N}\right)$	Mean, Var	Mean, Var	Mean, Var
