# Supplementary material for: Early prediction of antigenic transitions for influenza A/H3N2
Source: PLoS Comput Biol. 2020 Feb 18;16(2):e1007683. doi: 10.1371/journal.pcbi.1007683 (PMC7048310; doi:10.1371/journal.pcbi.1007683)
Supplement: S2 Table — The predictor variables are listed in the order by which they were selected using a forward selection algorithm. The coefficient estimate is the maximum and minimum coefficient (log-odds) from the five-fold cross validation of the final full-term model with the corresponding std. error. (PDF) [file pcbi.1007683.s010.pdf]

Surveillance Threshold (%)	Predictor Variable	Coefficient Estimate	Std. Error
1	$R_c/\langle R\rangle$	[2.61, 2.77]	[0.09,0.09]
	$\text{var}(R)$	[-0.54, -0.60)]	[0.06,0.06]
	$\langle R\rangle$	[0.30, 0.40]	[0.05-0.05]
	$k_c/\langle k\rangle$	[-0.20, -0.28)]	[0.06, 0.06]
	$\text{var}(\sigma_c)/\text{var}(S_{\text{eff}})$	[0.12, 0.16]	[0.05-0.06]
	$\text{var}(\beta_c)/\text{var}(\beta)$	[0.17, 0.24]	[0.05-0.05]
	$\text{var}(\sigma_c)$	[0.11, 0.19]	[0.05-0.06]
2	$R_c/\langle R\rangle$	[2.60, 2.71]	[0.09-0.10]
	$\text{var}(R)$	[-0.52, -0.57]	[0.06-0.07]
	$\langle R\rangle$	[0.28, 0.40]	[0.05-0.05]
	$k_c/\langle k\rangle$	[-0.27, -0.34]	[0.06-0.06]
	$\text{var}(\beta_c)/\text{var}(\beta)$	[0.32, 0.36]	[0.05-0.06]
	I/N	[-0.16, 0.18]	[0.06-0.06]
	$\text{var}(\sigma_c)$	[0.13, 0.19]	[0.06-0.06]
3	$R_c/\langle R\rangle$	[2.36, 2.43]	[0.09-0.10]
	$\text{var}(R)$	[-0.46, -0.57]	[0.07-0.07]
	$\langle R\rangle$	[0.39, 0.47]	[0.06-0.06]
	$k_c/\langle k\rangle$	[-0.32, -0.37]	[0.06-0.06]
	$\text{var}(\beta_c)/\text{var}(\beta)$	[0.26, 0.29]	[0.06-0.06]
	I	[-0.14, 0.21]	[0.06-0.06]
	$\max[[I_c//I_t]$	[0.11, 0.19]	[0.06-0.06]
4	$R_c/\langle R\rangle$	[2.53-2.75]	[0.10-0.11]
	$\text{var}(R)$	[-0.47, -0.63]	[0.07-0.08]
	$\langle R\rangle$	[0.30, 0.42]	[0.06-0.06]
	$k_c/\langle k\rangle$	[-0.22, -0.28]	[0.06-0.07]
	$\text{var}(\beta_c)/\text{var}(\beta)$	[0.30, 0.33]	[0.06-0.06]
	$\text{var}(\sigma_c)$	[0.15, 0.22]	[0.06-0.06]
	I	[-0.15, 0.17]	[0.06-0.06]
5	$R_c/\langle R\rangle$	[2.35, 2.58]	[0.11-0.12]
	$\text{var}(R)$	[-0.46, -0.59]	[0.08-0.08]
	$\langle R\rangle$	[0.31, 0.44]	[0.06-0.06]
	$k_c/\langle k\rangle$	[-0.25, -0.32]	[0.06-0.07]
	$\text{var}(\sigma_c)$	[0.17, 0.23]	[0.06-0.06]
	$\text{var}(\beta_c)/\text{var}(\beta)$	[0.14, 0.20]	[0.06-0.06]
	$\max[I_c/I_t]$	[0.11, 0.17]	[0.06-0.06]
6	$R_c/\langle R\rangle$	[2.36, 2.60]	[0.11-0.12]
	$\text{var}(R)$	[-0.60, -0.71]	[0.08-0.08]
	$\langle R\rangle$	[0.32, 0.43]	[0.06-0.06]
	$k_c/\langle k\rangle$	[-0.26, -0.17]	[0.06-0.06]
	$\text{var}(\beta_c)/\text{var}(\beta)$	[0.19, 0.23]	[0.06-0.06]
	$\text{var}(\sigma_c)$	[0.09, 0.21]	[0.06-0.07]
7	$R_c/\langle R\rangle$	[2.36, 2.59]	[0.12-0.13]
	$\text{var}(R)$	[-0.69, -0.78]	[0.08-0.08]
	$\langle R\rangle$	[0.32, 0.40]	[0.07-0.07]
	$k_c/\langle k\rangle$	[-0.25, -0.32]	[0.07-0.07]
	$\text{var}(\beta_c)/\text{var}(\beta)$	[0.17, 0.25]	[0.06-0.07]
8	$R_c/\langle R\rangle$	[2.22, 2.40]	[0.11-0.12]
	$\text{var}(R)$	[-0.51, -0.62]	[0.08-0.09]
	$\langle R\rangle$	[0.32, 0.42]	[0.07-0.07]
	$k_c/\langle k\rangle$	[-0.27, 0.34]	[0.07-0.07]
	$\text{var}(\beta_c)/\text{var}(\beta)$	[0.17, 0.25]	[0.07-0.07]
	$\max[I_c/I_t]$	[0.15, 0.29]	[0.07-0.07]
9	$R_c/\langle R\rangle$	[2.24, 2.45]	[0.12-0.13]
	$\text{var}(R)$	[-0.48, -0.63]	[0.09-0.10]
	$\langle R\rangle$	[0.30, 0.38]	[0.07.-0.07]
	$k_c/\langle k\rangle$	[-0.22, 0.31]	[0.07-0.07]
	$\max[I_c/I_t]$	[0.13, 0.25]	[0.07-0.07]
	$\text{var}(\beta_c)/\text{var}(\beta)$	[0.09, 0.24]	[0.07-0.07]
10	$R_c/\langle R\rangle$	[2.38, 2.55]	[0.13-0.14]
	$\text{var}(R)$	[-0.63, -0.74]	[0.09-0.09]
	$\langle R\rangle$	[0.27, 0.35]	[0.07-0.07]
	$k_c/\langle k\rangle$	[-0.18, -0.23]	[0.07-0.08]
	$\text{var}(\beta_c)/\text{var}(\beta)$	[0.14, 0.22]	[0.08-0.08]
	TMRCA	[-0.10, -0.21]	[0.08-0.08]
