# Supplementary material for: Early prediction of antigenic transitions for influenza A/H3N2
Source: PLoS Comput Biol. 2020 Feb 18;16(2):e1007683. doi: 10.1371/journal.pcbi.1007683 (PMC7048310; doi:10.1371/journal.pcbi.1007683)
Supplement: S3 Table — Model 1 shows the performance of the best-fit model using the actual values of relative fitness (relative growth rate) and competition (variance in the population growth rate) for clusters that reached the 5% surveillance thresholds (top two sections) and the 10% surveillance threshold (bottom two sections). Within each section, Model 2 substituted a time proxy for the fitness term and the absolute number of clusters that were growing for the competition term. Model 3 substituted a relative fold change for the fitness term and the population-wide variance in fold change for the competition term. t1 is when a focal cluster reaches the lower surveillance threshold (1%, 3%, 6%, 8%); t2 is when the same cluster reaches the higher surveillance threshold (5%, 10%) Performance metric values are the median across the five folds in cross-validation. Balanced accuracy measures the accuracy of the model, accounting for the imbalance in outcomes (i.e. number of transient versus established clusters) in the data set. In addition, to the terms included in the table, we tested the fold change of the dominant cluster from t1 and t2 as a predictor, but did not find that this term was a significant proxy in any model. (PDF) [file pcbi.1007683.s011.pdf]

**Surveillance Thresholds**	**Model**	Type	**Balanced Accuracy**	**AUC**	**PPV**	**Sensitivity**
5%	1. $R_c/\langle R\rangle$ + var(R)	Actual	0.78	0.88	0.81	0.89
1-5%	2. $\delta_c(t_1, t_2)$ + $N_{\Delta_j(t_1,t_2)>1}$	Proxy	0.57	0.71	0.65	0.93
1-5%	3. $\chi_c(t_1, t_2)$ + var($\Delta_j(t_1, t_2)$)	Proxy	0.50	0.58	0.61	0.99
5%	1. $R_c/\langle R\rangle$ + var(R)	Actual	0.78	0.88	0.81	0.89
3-5%	2. $\delta_c(t_1, t_2)$ + $N_{\Delta_j(t_1,t_2)>1}$	Proxy	0.56	0.67	0.64	0.95
3-5%	3. $\chi_c(t_1, t_2)$ + var($\Delta_j(t_1, t_2)$)	Proxy	0.50	0.58	0.61	0.99
10%	1.$R_c/\langle R\rangle$ + var(R)	Actual	0.78	0.88	0.81	0.89
6-10%	2.$\delta_c(t_1, t_2)$ + $N_{\Delta_j(t_1,t_2)>1}$	Proxy	0.70	0.78	0.74	0.87
6-10%	3.$\chi_c(t_1, t_2)$ + var($\Delta_j(t_1, t_2)$)	Proxy	0.59	0.67	0.66	0.95
10%	1. $R_c/\langle R\rangle$ + σ_R	Actual	0.78	0.88	0.81	0.89
8-10%	2. $\delta_c(t_1, t_2)$ + $N_{\Delta_j(t_1,t_2)>1}$	Proxy	0.63	0.72	0.68	0.95
8-10%	3. $\chi_c(t_1, t_2)$ + var($\Delta_j(t_1, t_2)$)	Proxy	0.58	0.70	0.65	0.97
