# Supplementary material for: Early prediction of antigenic transitions for influenza A/H3N2
Source: PLoS Comput Biol. 2020 Feb 18;16(2):e1007683. doi: 10.1371/journal.pcbi.1007683 (PMC7048310; doi:10.1371/journal.pcbi.1007683)
Supplement: S4 Table — Terms are listed in the order they were added to the model through forward-selection. (PDF) [file pcbi.1007683.s012.pdf]

Months Ahead	Predictors	AUC	PPV	Sensitivity
3	f_c<br>R_c<br>$\langle R \rangle$<br>$\beta_c/\langle \beta \rangle$<br>$\text{var}(\beta_c)/\text{var}(\beta)$	0.93	0.90	0.96
6	f_c<br>R_j<br>$\langle R \rangle$<br>$\beta_c/\langle \beta \rangle$<br>$\text{var}(R)$<br>$\text{var}(\beta_c)/\text{var}(\beta)$<br>$I$	0.93	0.89	0.89
9	R_c<br>f_c<br>$\langle R \rangle$<br>$\beta_c/\langle \beta \rangle$<br>$\text{var}(R)$<br>$\text{var}(\beta_c)/\text{var}(\beta)$<br>tMRCA	0.93	0.84	0.91
12	R_j<br>f_c<br>$\langle R \rangle$<br>$\text{var}(R)$<br>$\beta_c/\langle \beta \rangle$<br>$\text{var}(\beta_c)/\text{var}(\beta)$	0.92	0.81	0.87
