# Supplementary material for: Early prediction of antigenic transitions for influenza A/H3N2
Source: PLoS Comput Biol. 2020 Feb 18;16(2):e1007683. doi: 10.1371/journal.pcbi.1007683 (PMC7048310; doi:10.1371/journal.pcbi.1007683)
Supplement: S5 Table — Terms are listed in the order they were added to the model through forward-selection. The Adjusted R2 and Root Mean Squared Error (RMSE) were measured on a testing data set of 5 random time samples over a 25-year period. (PDF) [file pcbi.1007683.s013.pdf]

**Months Ahead**	**Predictors**	R^2 **Adjusted**	**RMSE (log)**
3	f_c<br>R_c<br>$\langle R \rangle$<br>$\beta_c/\langle \beta \rangle$	0.84	0.36
6	f_c<br>R_c<br>$\langle R \rangle$<br>$\beta_c/\langle \beta \rangle$<br>$\mathrm{var}(R)$	0.74	0.51
9	R_c<br>f_c<br>$\langle R \rangle$<br>$\mathrm{var}(R)$<br>$\beta_c/\langle \beta \rangle$<br>$\mathrm{var}(\beta_c)/\mathrm{var}(\beta)$	0.66	0.65
12	R_j<br>f_c<br>$\langle R \rangle$<br>$\mathrm{var}(R)$<br>$\mathrm{var}(\beta_c)/\mathrm{var}(\beta)$<br>$\beta_c/\langle \beta \rangle$	0.57	0.77
