# Supplementary material for: Early prediction of antigenic transitions for influenza A/H3N2
Source: PLoS Comput Biol. 2020 Feb 18;16(2):e1007683. doi: 10.1371/journal.pcbi.1007683 (PMC7048310; doi:10.1371/journal.pcbi.1007683)
Supplement: S6 Table — Values were taken at the first two sample points a focal antigenic cluster was recorded. All viruses within a cluster have the same number of epitope mutations, ρ. Symbols for quantities are consistent with S1 and S3 Tables. (PDF) [file pcbi.1007683.s014.pdf]

Candidate Predictor	Formula	Metric Type
Frequency at t	$f_c(t)$	
Number of Epitope Mutations in Cluster c	ρ_c	
Relative Number of Epitope Mutations at t	$\frac{\rho_c * f_c(t)}{\frac{1}{N_c}\sum_{j=1}^{N_c} \rho_j * f_j(t)}$	Fitness
Frequency Fold Change	$\Delta_c(t_1, t_2) = \frac{f_c(t_2)}{f_c(t_1)}$	
Relative Fold Change	$\chi_c(t_1, t_2) = \frac{\Delta_c(t_1,t_2)}{\frac{1}{N_c}\sum_{j=1}^{N_c} \Delta_j(t_1,t_2)}$	
Growth Rate	$\frac{\Delta_c(t_1,t_2)}{t_1 - t_2}$	
Average Cluster Frequency at t	$\frac{1}{N_c}\sum_{j=1}^{N_c} f_j(t)$	
Variance in Cluster Frequency at t	$\mathrm{var}(f_c(t))$	
Population Mean Fold Change	$\frac{1}{N_c}\sum_{j=1}^{N_c} \Delta_j(t_1, t_2) * f_j(t)$	
Variance in Fold Change	$\mathrm{var}(\Delta_j(t_1, t_2))$	
Population Mean Number of Epitope Mutations	$\frac{1}{N_c}\sum_{j=1}^{N_c} \rho_j * f_c(t)$	Competition
Number of cocirculating clusters at t	$N_c(t)$	
Number of clusters lost from t_1 to t_2	$\lvert C(t_2) \notin C(t_1)\rvert$	
Percent of clusters lost from t_1 to t_2	$\frac{\lvert C(t_2) \notin C(t_1)\rvert}{N_c(t_1)}$	
