# Supplementary material for: Early prediction of antigenic transitions for influenza A/H3N2
Source: PLoS Comput Biol. 2020 Feb 18;16(2):e1007683. doi: 10.1371/journal.pcbi.1007683 (PMC7048310; doi:10.1371/journal.pcbi.1007683)
Supplement: S7 Table — *All models marked have the same F1 score and are listed in order of descending AUC score. The AUC is the average of the AUC from the testing data, while the PPV and Sensitivity are measured at the threshold that maximizes the F1 score. For all empirical models, the frequency of the focal cluster, fc, at either t1 or t2 was the best fitness measure. The competition term either captured the average or variance in the frequency of competing clusters or how the absolute number of competing changes changed from t1 to t2. (PDF) [file pcbi.1007683.s015.pdf]

| **Rank** | **Model** | **AUC** | **PPV** | **Sensitivity** |
|---|---|---|---|---|
| 1. | $f_c(t_2) + \lvert C(t_2) \notin C(t_1) \rvert$ | 0.756 | 0.500 | 0.556 |
| 2.* | $f_c(t_2) + \frac{1}{N_c}\sum_{j=1}^{N_c} f_j(t_1)$ | 0.813 | 0.667 | 0.333 |
| 3.* | $f_c(t_2) + \mathrm{var}(f_c(t_1))$ | 0.807 | 0.667 | 0.333 |
| 4.* | $f_c(t_2) + \mathrm{var}(\Delta_j(t_1, t_2))$ | 0.712 | 0.667 | 0.333 |
| 5.* | $f_c(t_2) + \frac{1}{N_c}\sum_{j=1}^{N_c} \Delta_j(t_1, t_2) * f_j(t_2)$ | 0.708 | 0.667 | 0.333 |
| 6.* | $f_c(t_2) + \frac{1}{N_c}\sum_{j=1}^{N_c} f_j(t_2)$ | 0.644 | 0.667 | 0.333 |
| 7. | $f_c(t_1) + \mathrm{var}(f_c(t_1))$ | 0.782 | 0.611 | 0.333 |
| 8. | $f_c(t_1) + \lvert C(t_2) \notin C(t_1) \rvert$ | 0.741 | 0.340 | 0.556 |
| 9. | $f_c(t_1) + \frac{1}{N_c}\sum_{j=1}^{N_c} f_j(t_2)$ | 0.637 | 0.611 | 0.333 |
| 10. | $f_c(t_1) + \mathrm{var}(f_c(t_2))$ | 0.626 | 0.611 | 0.333 |
